# Optimization of Electrolytes for High-Performance Aqueous Aluminum-Ion Batteries

**DOI:** 10.1021/acsami.1c23278

**Published:** 2022-05-27

**Authors:** Andinet Ejigu, Lewis W. Le Fevre, Amr Elgendy, Ben F. Spencer, Carlo Bawn, Robert A. W. Dryfe

**Affiliations:** † Dept. of Chemistry, University of Manchester, Oxford Road, Manchester M13 9PL, U.K.; ‡ Henry Royce Institute, University of Manchester, Oxford Road, Manchester M13 9PL, U.K.; § Dept. of Materials, University of Manchester, Oxford Road, Manchester M13 9PL, U.K.

**Keywords:** aluminum-ion battery, water-in-salt, aluminum bis(trifluoromethanesulfonyl)imide, Al−Zn alloy anode, MnO_2_ cathode, electrodeposition

## Abstract

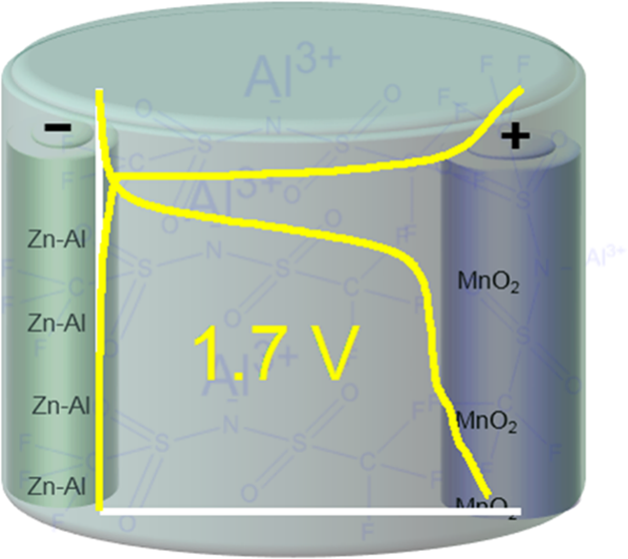

Aqueous rechargeable batteries based on aluminum chemistry have become the focus of immense research interest owing to their earth abundance, low cost, and the higher theoretical volumetric energy density of this element compared to lithium-ion batteries. Efforts to harness this huge potential have been hindered by the narrow potential window of water and by passivating effects of the high-electrical band-gap aluminum oxide film. Herein, we report a high-performing aqueous aluminum-ion battery (AIB), which is constructed using a Zn-supported Al alloy, an aluminum bis(trifluoromethanesulfonyl)imide (Al[TFSI]_3_) electrolyte, and a MnO_2_ cathode. The use of Al[TFSI]_3_ significantly extends the voltage window of the electrolyte and enables the cell to access Al^3+^/Al electrochemistry, while the use of Zn–Al alloy mitigates the issue of surface passivation. The Zn–Al alloy, which is produced by in situ electrochemical deposition, obtained from Al[TFSI]_3_ showed excellent long-term reversibility for Al electrochemistry and displays the highest performance in AIB when compared to the response obtained in Al_2_(SO_4_)_3_ or aluminum trifluoromethanesulfonate electrolyte. AIB cells constructed using the Zn–Al|Al[TFSI]_3_|MnO_2_ combination achieved a record discharge voltage plateau of 1.75 V and a specific capacity of 450 mAh g^–1^ without significant capacity fade after 400 cycles. These findings will promote the development of energy-dense aqueous AIBs.

## 1 Introduction

The increased demand for improved and low-cost rechargeable electrochemical energy storage (EES) devices is being driven by the expanding quantity of consumer electronic devices and electric cars, as well as the expansion of energy derived from intermittent renewable sources. A novel EES system that employs readily available raw materials with high energy densities and safety is required to meet future energy demand. To date, lead-acid and lithium-ion batteries (LIBs) have dominated the battery market. In particular, LIBs have been the most commonly used EES systems for portable electronic devices and are now making substantial inroads into the vehicle market.^1^ While humanity’s reliance on LIBs is increasing in our daily life, its major raw materials including lithium, cobalt, and nickel are predicted to be exhausted within the next decade.^2^ In addition, those raw materials are concentrated in a few regions of the world while demand is growing everywhere.^1,3^ As a result, there is an urgent need for the development of alternative battery chemistry that utilizes earth-abundant raw materials.

Aluminum-based batteries are regarded as the most promising alternatives due to their wide availability, Al being the third most abundant element in the Earth’s crust, low cost, low intrinsic flammability, and providing ease of processing and recycling. Aluminum has a high theoretical specific capacity per unit mass (2981 mAh g^–1^) and the highest capacity per unit volume (8056 mAh cm^–3^) owing to its three-electron reduction process (Al^3+^/Al).^4^ These favorable properties have long attracted the attention of researchers with the first use of Al in an electrochemical cell dating back to the 1850s.^5^ The Al negative electrode has been investigated in both primary and secondary cells employing either aqueous or nonaqueous electrolytes although none of the cells were commercialized to date.^6^ Of the electrolytes studied thus far at room temperature, the chloroaluminate ionic liquids are the electrolytes that support the reversible Al^3+^/Al electrochemistry with a high Coulombic efficiency (>99.5%).^7−10^ A prototype “Al-ion battery” constructed using a chloroaluminate electrolyte, an Al foil negative electrode, and carbon-based cathodes showed stable ultrafast charge–discharge processes with a discharge voltage of ∼2.0 V and a specific capacity of ∼80 mAh g^–1^.^8,11−13^ The high cost, air sensitivity, and severe corrosiveness toward conventional current collectors/battery packing materials created complexity in battery design when using chloroaluminate electrolytes. In addition, the cell based on this electrolyte exhibited a low energy density due to^4,11,14^ (i) the intercalation process involving only a one-electron ([AlCl_4_]^−^) transfer instead of intercalating Al^3+^ through a three-electron-transfer process; (ii) a limitation due to a lack of suitable positive electrode materials that can accommodate the large [AlCl_4_]^−^ species with good capacity; and (iii) lack of sufficient electroactive reactant within the electrolyte ([AlCl_4_]^−^ and [Al_2_Cl_7_]^−^) that limits the capacity of the negative electrode as the anodic half-reaction requires eight Al atoms per three electrons instead of one Al atom per three electrons.

The realization of reversible Al negative electrode electrochemistry using aqueous solution is hindered by several fundamental factors including the passivating oxide film, negative electrode corrosion, and the narrow electrochemical window of water, i.e., water decomposes to hydrogen gas well before Al^3+^ reduction occurs (*E*° = Al^3+^/Al = −1.7 V vs SHE in contrast to H_2_O/H_2_ = 0.0 to −0.8 V vs SHE depending on the pH). Furthermore, Al readily forms a high-band-gap passivating oxide film when exposed to air.^5^ This oxide coating makes the Al surface inaccessible for redox reactions or requires a high driving overpotential to transport ions through the oxide film. However, due to the narrow electrochemical window of water, this potential can easily exceed the thermodynamic limit of water reduction resulting in continuous electrolyte degradation through hydrogen evolution. Zhao et al. demonstrated that the immersion of Al electrode in chloroaluminate ionic liquid could erode the oxide film and create an artificial solid electrolyte interface (SEI) that is stable at ambient atmosphere.^15^ Moreover, the interface protects the Al surface from further passivation. The treated electrodes exhibited good reversibility in symmetric Al cells when combined with an aluminum trifluoromethanesulfonate (Al[OTF]_3_) electrolyte. Using this pretreated Al negative electrode and a MnO_2_ positive electrode with (Al[OTF]_3_) electrolyte, a discharge voltage of 1.4 V and a high specific capacity of 380 mAh g^–1^ were reported. Alternatively, Yan et al. recently showed that a Zn substrate can successfully support the reversible electrodeposition of Al^3+^ from aqueous Al[OTF]_3_ electrolytes through the formation of a Zn–Al alloy.^16^ The addition of zinc to Al, to form an alloy, is known to mitigate the passivation film, reduce the self-corrosion of Al, and may increase the overpotential for the competing hydrogen evolution reaction.^17^ The Zn–Al|MnO cell in Al[OTF]_3_ electrolytes achieved the highest discharge voltage plateau of 1.6 V and a specific capacity of 460 mAh g^–1^.

The hydrogen evolution side reaction is still the main factor hindering the practical development of AIBs due to the Lewis acidity of aqueous Al-based electrolytes (pH < 1) coupled with the high negative standard potential of Al^3+^/Al. Recent research from aqueous lithium-ion,^18−20^ sodium-ion,^21−23^ magnesium-ion,^24^ and zinc-ion batteries^25,26^ showed that the choice of the electrolytic anions and their concentration significantly impact the electrochemical window of water. In particular, water-in-salt electrolytes based on perfluorinated sulfonylimide anions such as bis(trifluoromethanesulfonyl)imide([TFSI]) displayed the highest potential stability and minimal dendrite formation at the metallic negative electrode and formed an effective interfacial layer on the surface of the electrode.^25,27,28^ Dubouis et al. demonstrated that the hydroxides generated during initial hydrogen evolution react with the [TFSI] anion to form an SEI that prevents further water reduction at the negative electrode.^29^ An alternative mechanism for the SEI formation was the preferential reduction of [TFSI] anion relative to water reduction leading to the formation of an anion-derived SEI on the negative electrode surface that contributes to the wider electrochemical window stability.^18,30^ These studies strongly indicate that the use of [TFSI]-based electrolytes in AIBs mitigates the critical challenge of the parasitic hydrogen evolution reaction on the negative electrode. To the best of our knowledge, there is no prior report on the use of an aluminum bis(trifluoromethanesulfonyl)imide (Al[TFSI]_3_) electrolyte for aqueous AIBs, aside from their use as conducting additives in an organic solvent to suppress the anodic dissolution of the aluminum current collectors.^31,32^ Herein, we synthesize Al[TFSI]_3_ via a simple ion-exchange reaction and use it as an electrolyte for aqueous AIBs. We show that the electrochemical window of aqueous Al[TFSI]_3_ is at least 1.0 V larger than Al[OTF]_3_ or Al_2_(SO_4_)_3_ (aq) electrolytes at comparable salt concentration. The use of Al[TFSI]_3_ electrolytes using Zn–Al negative electrode and MnO_2_ positive electrode achieved the highest discharge voltage plateau (1.75 V) reported to date with a high specific capacity of 450 mAh g^–1^. Moreover, we also show that the high performance obtained using Al[TFSI]_3_ electrolytes is attributed to the facile Al^3+^/Al–Zn_*X*_ negative electrode electrochemistry, accessibility of a larger potential window without electrolyte decomposition. A combination of electron microscopy and spectroscopic techniques has been used to gain these mechanistic insight processes for each electrolyte as well as the electrode reactions. Finally, we determine the optimum Al[TFSI]_3_ concentration for good cell performance and present data demonstrating the high stability of cell cycling.

## 2 Results and Discussion

### 2.1 Electrolyte Optimization

The Al[TFSI]_3_ salt was prepared by reacting neat trifluoromethanesulfonimide acid [(CF_3_SO_2_)_2_NH] with anhydrous AlCl_3_ under an Ar-filled glovebox. This reaction also generates hydrogen chloride gas as a byproduct, which was removed by heating in a vacuum oven or, preferably, in a Schlenk line. The complete formation of Al[TFSI]_3_ and the removal of the HCl impurity are confirmed using X-ray photoelectron spectroscopy (XPS) and nuclear magnetic resonance spectroscopy (NMR). As shown in Figure S1, XPS revealed the absence of Cl and the presence of Al as well as other elements associated with the [TFSI] anion, which demonstrates the complete reaction to form Al[TFSI]_3_. Moreover, ^1^H NMR showed the absence of the acidic proton from the reactant and the presence of ^27^Al and ^19^F confirming the formation of the Al[TFSI]_3_ (Figure S1C–E).

**Figure 1:**
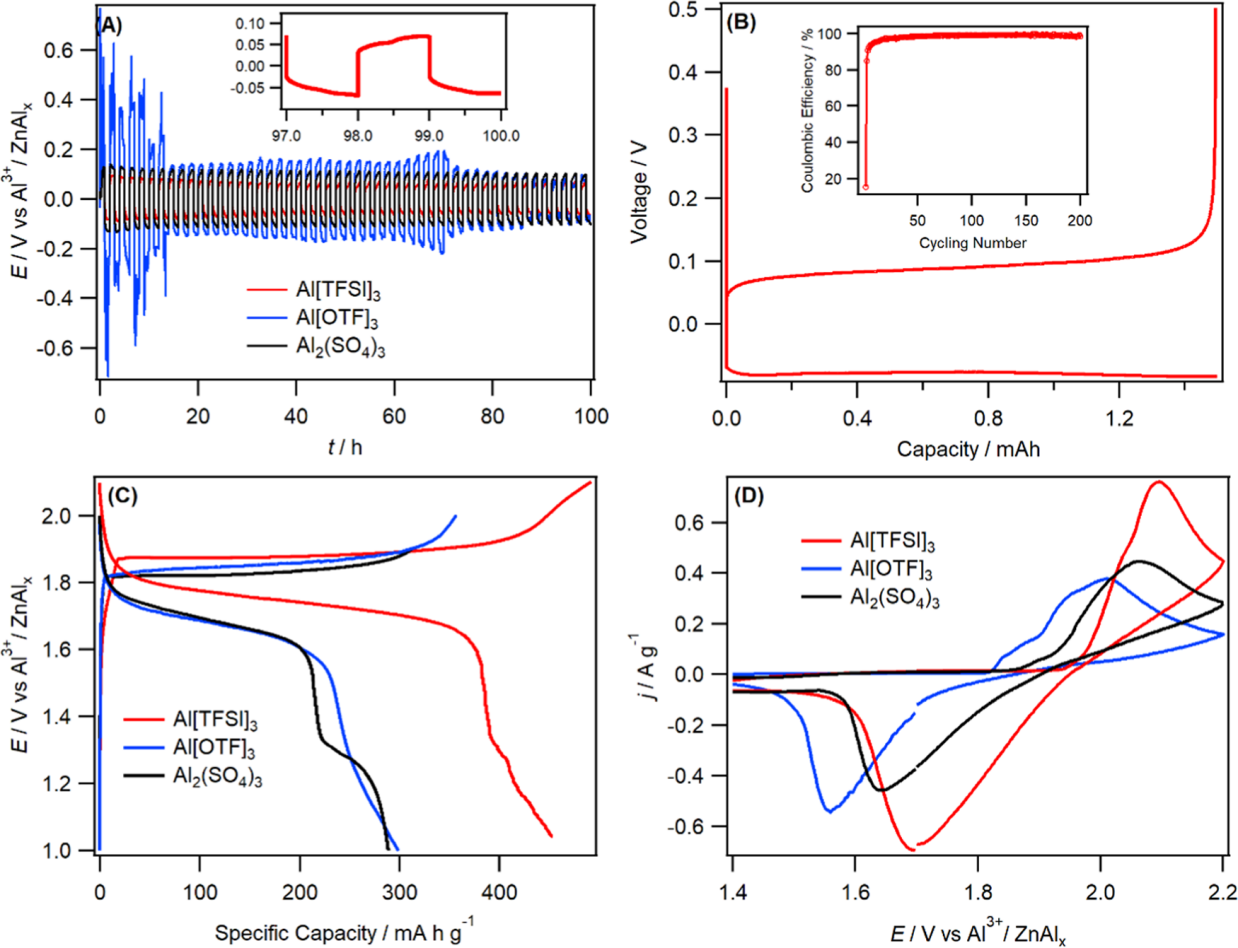
(A) Galvanostatic charge–discharge curve obtained using a symmetrical Zn|Zn cell in 3m Al[TFSI]_3_, 2m Al[OTF]_3_, and Al_2_(SO_4_)_3_ electrolytes at 0.2 mA cm^–2^. The inset shows the last 3 h cycles obtained in 3m Al[TFSI]_3_, (B) charge–discharge vs capacity profiles measured during Al plating/stripping at the Zn/carbon cloth cell using 3m Al[TFSI]_3_ at an applied current density of 2 mA cm^–2^. The inset shows the Coulombic efficiency evolution with cycling obtained using 3m Al[TFSI]_3_. (C) Galvanostatic charge–discharge curves (second cycle) vs capacity obtained at a current density of 25 mA g^–1^ using Zn–Al*_X_* and MnO_2_ positive electrode in 3m Al[TFSI]_3_, 2m Al[OTF]_3_, and 2m Al_2_(SO_4_)_3_ electrolytes, (D) Cyclic voltammograms recorded at 0.1 mV s^–1^ in the electrolyte given (see the figure) using coin cells constructed from MnO_2_ positive electrodes and a Zn–Al*_X_* foil The gravimetric current is calculated from the mass of the cathode.

The suitability of the Al[TFSI]_3_ electrolyte for AIBs was then assessed by various electrochemical methods and its cell performance compared to those of Al[OTF]_3_ and Al_2_(SO_4_)_3_ electrolytes. First, the electrochemical deposition and stripping of Al^3+^, as well as the long-term stability, were compared in a symmetrical cell using Zn foil as a substrate. We should note that we used 2m Al[OTF]_3_ as a comparison as Yan et al. showed that 2m is the optimum concentration in AIB performance.^16^ In addition, 2m Al_2_(SO_4_)_3_ was used, as this is its maximum solubility limit in water. Figure 1A shows the charge–discharge curve obtained using the three electrolytes in a symmetrical Zn|Zn cell at 0.2 mA cm^–2^. In the initial charge–discharge cycle, Al^3+^ is expected to deposit at the negative Zn electrode while the stripping of Zn to Zn^2+^ occurs at the positive electrode. The codeposition of Zn^2+^ along with Al^3+^ is expected to occur during subsequent charge–discharge to form a Zn–Al_*X*_ alloy (where “*x*” represents the stoichiometry of Al electrodeposited).^16^ Cyclic voltammetry was used in a three-electrode cell setup to confirm the electrodeposition of Al^3+^ on a Zn working electrode from the Al[TFSI]_3_ electrolyte. As shown in Figure S2A, during the first potential scan from an initial potential of −1.4 V *vs* Ag|AgCl, a reductive current started to flow and peaked at about −1.9 V due to the electrodeposition of Al. In the return scan, a large sharp current due to the stripping of Al was observed. The co-oxidation of Zn also occurs if the electrode potential is intentionally scanned all the way to zinc stripping potential during this oxidative scan as an additional reductive peak was seen at −1.25 V due to the codeposition of Zn in the subsequent scan. However, the bulk oxidation of Zn^2+^ is less likely to occur during battery operation given that the standard redox potential of Zn^2+^/Zn is too positive when compared to the anode reaction (Zn–Al_*X*_ alloy formation). Indeed, determination of the content of Zn in the Al[TFSI]_3_ electrolyte solution after several charge–discharge processes using inductively coupled plasma atomic emission spectroscopy detected a trace amount of Zn^2+^ (0.3%). Characterization of both the positive and negative electrodes obtained in Al[TFSI]_3_ after cycling using X-ray diffraction (XRD) and energy-dispersive X-ray spectroscopy (EDX) revealed the formation of Zn–Al_*X*_ alloy (Figures S3 and S4). EDX data showed the electrodeposition of Al, while the X-ray diffraction (XRD) pattern shows peaks that match the Zn reference substrate. However, the positions of the diffraction pattern are shifted to a lower angle compared to pure Zn, indicating the alloying effect of Zn with Al.^33,34^ Yan et al. also observed similar behavior when analyzing the XRD pattern obtained from electrodeposition of Al^3+^ on Zn foil from the Al[OTF]_3_ electrolyte.^16^ In addition, there were no detectable peaks in the XRD pattern due to pure Al even at a high deposition capacity, suggesting that the bulk electrodeposition of Al on the Zn surface is not observed. It was shown that the signal due to the face-centered cubic Al phase was only visible when the alloy contained more than 50% of Al.^35^ In our case, the atomic concentration of the electrodeposited Al was only about 5% (see the next section). Note that this percentage was obtained using a MnO_2_ cathode at a loading of about 2 mg cm^2^. In theory, the amount of Al on the Zn can be increased by increasing the MnO_2_ amount on the current collector or by using a thin Zn surface in the range of a tenth of micron to harness the full potential of Al capacity. The XRD patterns of the anode (obtained in Zn|MnO_2_ cell) show that as the deposition capacity of Al increases, the diffraction peaks of this alloy monotonically shift toward a low angle, indicating that the lattice constant is dependent on the alloy composition (Figure S4C). The charge–discharge curve shows the lowest potential separation for Al^3+^ deposition and oxidation in Al[TFSI]_3_ (red line) and the largest in Al[OTF]_3_ (blue line), indicating that the rate of the Al^3+^/Al deposition reaction is fastest in Al[TFSI]_3_ electrolytes. The Al[TFSI]_3_ electrolyte also showed a stable overpotential (0.15 V) throughout the 100 h cycles when compared to Al[OTF]_3_, which showed an overpotential of over 1.0 V during the initial 15 h cycles. Furthermore, the Coulombic efficiency (CE) of the alloy formation and stripping in the Al[TFSI]_3_ electrolyte was 99.3% (Figure 1B), whereas it was less than 30% in the Al_2_(SO_4_)_3_ electrolyte (Figure S2B), implying that hydrogen evolution reaction is greatly reduced in the [TFSI]-based anion. We also noted that CE increased initially with increasing charge–discharge cycling, presumably due to the continual formation of a stable interface. On the other hand, even in Al[TFSI]_3_ electrolytes (Figure S5A), the electrochemistry of the symmetrical Al cell displayed an overpotential of over 6.0 V after 20 cycles, and subsequently, the cell failed due to the passivating oxide layer and hydrogen evolution side reaction.^36^ These data demonstrate that the Zn substrate can successfully support the reversible deposition of Al^3+^ from aqueous Al^3+^-based electrolytes with high efficiency to form Zn–Al*_X_* surface alloy.

Figure 1C shows the comparison of the charge–discharge curve obtained for the AIBs full cell with the three Al-based electrolytes using an α-MnO_2_ cathode. The Al[TFSI]_3_ electrolyte displayed the highest performance in terms of discharge capacity and average voltage plateau (1.75 V), and the rate capability is shown in Figure S5C. Specific capacities of 450, 300, and 290 mAh g^–1^ were obtained in Al[TFSI]_3_, Al[OTF]_3_, and Al_2_(SO_4_)_3_, respectively. In addition, the average discharge voltage plateau in Al[TFSI]_3_ is larger than the other two electrolytes by ∼100 mV, in agreement with the cyclic voltammetry (CV) response, which shows an average potential of 1.9 V when compared to 1.8 V for Al[OTF]_3_ (Figure 1D). The CV response of the full cell also showed that the rate of Al^3+^/Al reaction is fastest when the Al[TFSI]_3_ electrolyte was used, as characterized by a lower peak-to-peak separation of 0.4 V compared to 0.46 V in the Al[OTF]_3_ electrolyte. The average voltage plateau obtained in Al[TFSI]_3_ electrolytes is, to the best of our knowledge, the highest discharge voltage among all of the reported AIBs based on a manganese oxide cathode. Yan et al.^16^ reported a voltage plateau of 1.6 V using Zn–Al|Al[OTF]_3_|MnO cell, while others reported plateaux below 1.4 V when using a pure Al negative electrode and manganese oxide cathodes.^15,37^ We should also point out that the use of premade Zn–Al_*X*_ is not essential as *in situ* Al^3+^ deposition occurs on the Zn negative electrode, while the insertion of H^+^/Al^3+^ occurs at the positive electrode (α-MnO_2_) during the cell charge–discharge process. In fact, a significant decrease in cell performance was seen when premade Zn–Al_*X*_ was used as the negative electrode (see Figure S6), characterized by lower discharge voltage (<1.6 V) and reduced specific capacity.

### 2.2 Structural and Compositional Characterization of Electrodes and Electrolytes

**Figure 2:**
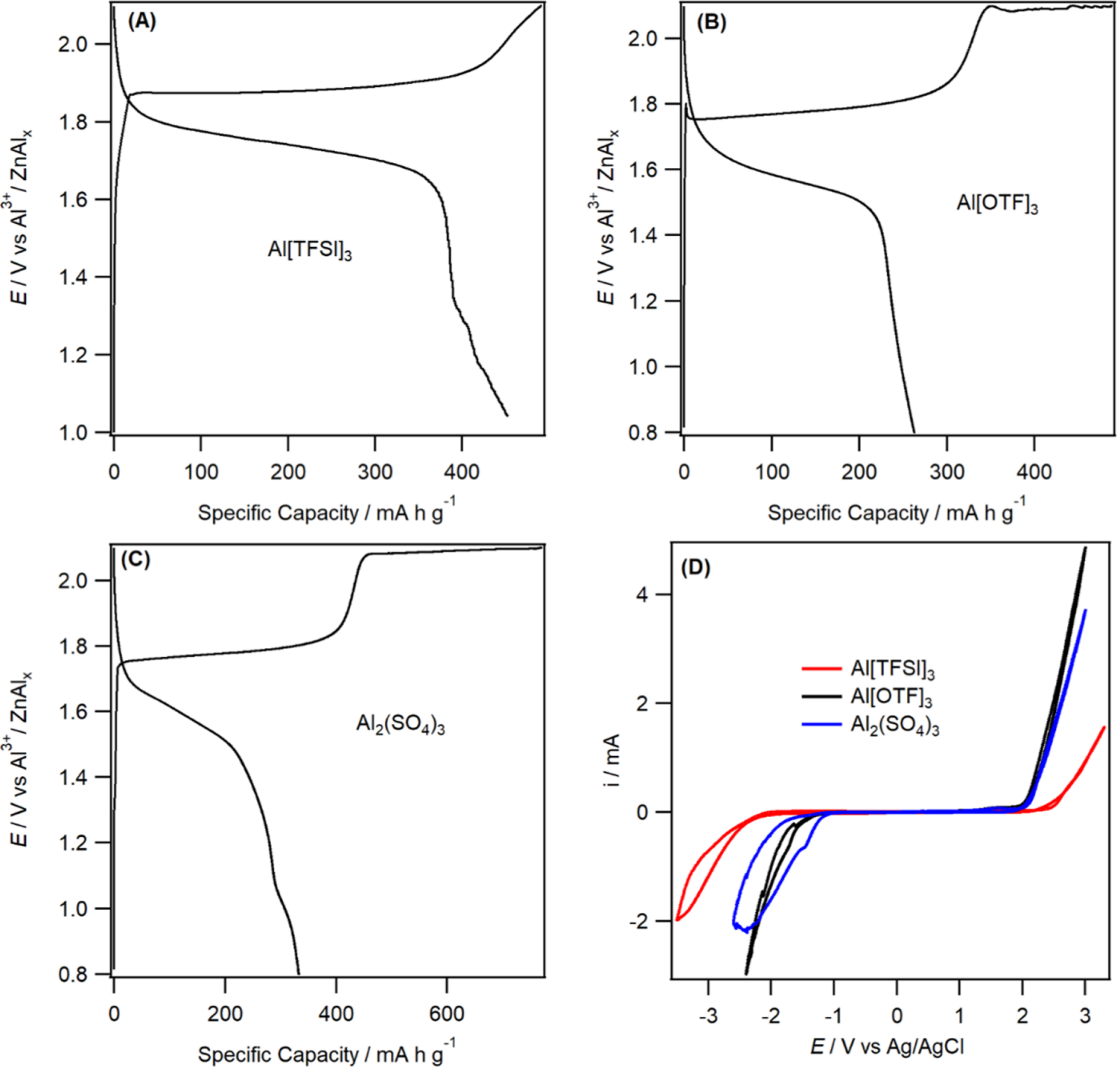
Galvanostatic charge–discharge curves vs capacity obtained at a current density of 25 mA g^–1^ using Zn–Al*_X_* and MnO_2_ positive electrodes in (A)3m Al[TFSI]_3_, (B) 2m Al[OTF]_3_, (C) 2m Al_2_(SO_4_)_3_ electrolytes, and (D) CVs recorded at a 3 mm diameter glassy carbon electrode in a three-electrode configuration at 10 mV s^–1^ using 3m Al[TFSI]_3_, 2m Al[OTF]_3_, and 2m Al_2_(SO_4_)_3_ electrolytes.

**Figure 3:**
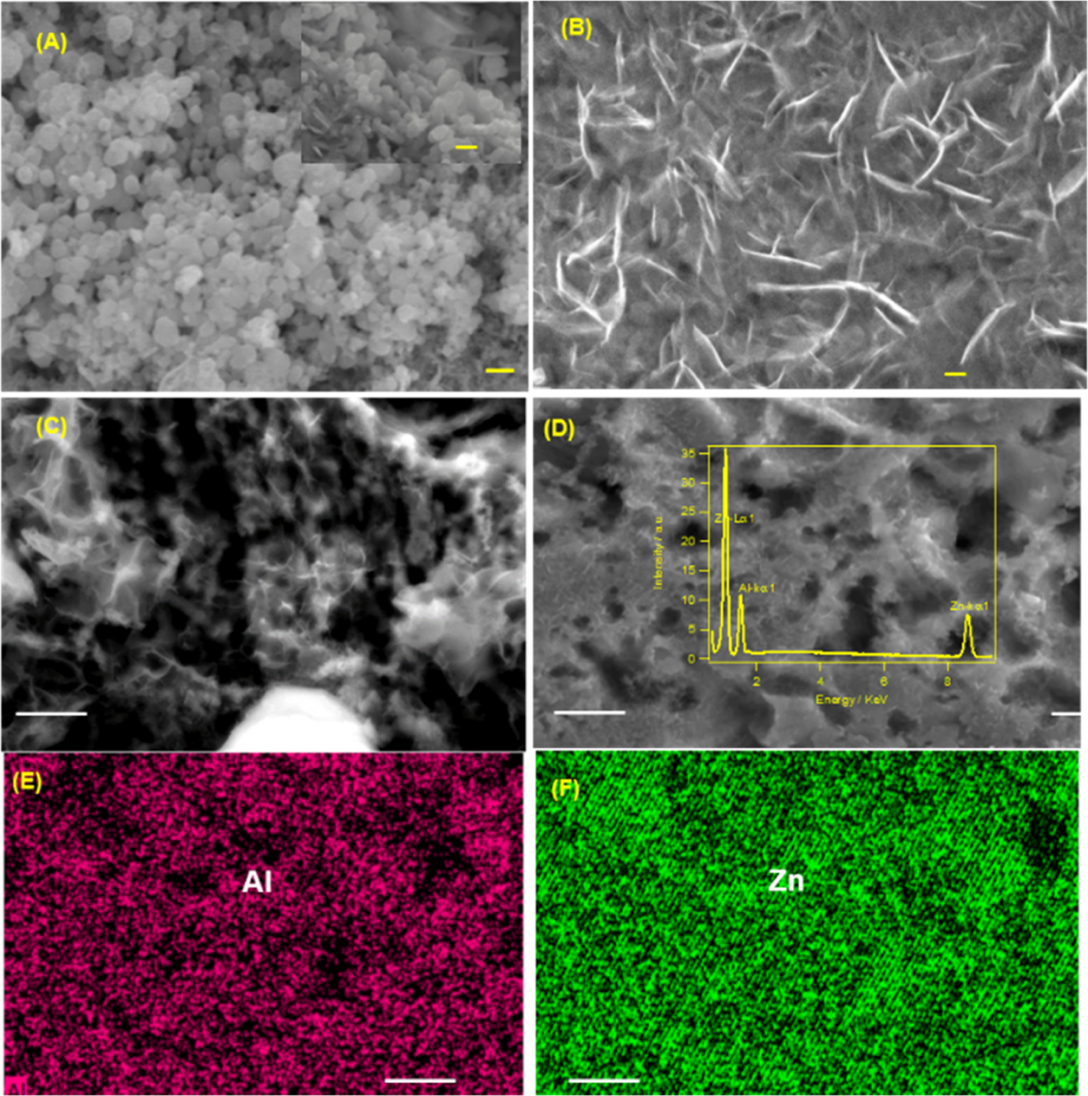
SEM images of the Zn–Al*_X_* electrodes after charging in full cell using Zn foil and MnO_2_ positive electrodes in (A) 3m Al[TFSI]_3_; the inset shows the high-magnification (B) 2m Al[OTF]_3_ and (C) 2m Al_2_(SO_4_)_3_. (D) SEM image of the Zn–Al*_X_*, where EDX mapping was taken, and the inset shows the EDX spectrum of the mapping showing elemental Al and Zn. (E) and (F) Elemental mapping of the Zn–Al*_X_* electrode. The scale bars in panels (A–C) are 1 μm (inset is 0.5 μm) and 4 μm in panels (D–F).

There are a few reasons for the enhanced performance seen with the Al[TFSI]_3_ electrolyte. First, as discussed in the context of Figure 1A, the kinetics of Al^3+^ deposition and stripping on the Zn substrate are more facile than with Al[OTF]_3_ or Al_2_(SO_4_)_3_ electrolyte. Second, the electrochemical window of the Al[TFSI]_3_ electrolyte is the highest, when compared to Al[OTF]_3_ or Al_2_(SO_4_)_3_, enabling the cell to be charged to a higher voltage. As shown in Figure 2A, the cell with the Al[TFSI]_3_ electrolyte can be charged to 2.1 V without electrolyte degradation and the CE of the cell was over 93%. A significant amount of electrolyte degradation occurred in Al[OTF]_3_ and Al_2_(SO_4_)_3_ electrolytes when the cells were charged to 2.1 V, characterized by a dramatic decrease in CE to below 50% (Figure 2B,C). Indeed, the overall electrochemical window characterization of the three electrolytes using a glassy carbon disk electrode also revealed that Al[TFSI]_3_ showed the highest electrochemical window of 4.3 V when compared to that of 3.2 V in Al[OTF]_3_ and 3.0 V in Al_2_(SO_4_)_3_ (Figure 2D). It is also interesting to note that the larger increase in the electrochemical window of Al[TFSI]_3_ is obtained by hindering hydrogen evolution, rather than oxygen evolution, when compared to the other two electrolytes, as seen also by the CE data in Figure 1B and S2B. The high electrochemical oxidative stability of the [TFSI]^−^ anion is known from research in various aqueous battery systems.^18,24^ The reduction of the [TFSI]^−^ anion is believed to form a passivating solid layer interface that increases the overall electrochemical window.^38^ The surface passivation of [TFSI]^−^ was also shown by a previous study, which compared the adsorption of [TFSI]^−^ and [OTF]^−^ on Pt from neat ionic liquids.^39^ Finally, the surface morphology of the cycled negative electrode from the full cell also revealed some insight into the different performance. Figure 3A,C shows the scanning electron microscopy (SEM) images of the negative electrode obtained after charge–discharge cycling in the Zn–Al_*X*_|MnO_2_ full cell. The Zn–Al_*X*_ deposits obtained using Al[TFSI]_3_ showed uniform and well-defined nanoparticles (Figure 3A). EDX mapping revealed that Al was uniformly deposited across the Zn foil with a 5% average atomic percentage (Figure 3D,F). In contrast, the deposits obtained from Al[OTF]_3_ and Al_2_(SO_4_)_3_ showed a high-surface-area honeycomb-like structure that can be prone to form dendrites over multiple cycles (Figure 3B,C).

The solvation structure of Al^3+^, hydrogen evolution side reaction, interaction between water molecules and electrolyte anions, as well as the overall voltage applied to the cell may affect the morphology of the Al electrodeposits. For example, the upper cutoff voltage for Al[TFSI]_3_ is 2.1 V compared to that of 2.0 V for Al[OTF]_3_ or Al_2_(SO_4_)_3_. This voltage difference together with the slow Al^3+^/Al electrode kinetics in the latter electrolytes may produce different growth morphologies. The hydrogen evolution side reaction also affects the growth of electrodeposited nanostructures. In fact, it was shown that the honeycomb morphology is the preferred growth pattern when hydrogen codeposition occurs during metal electrodeposition.^40^ In addition, the solvation structure of the electrolytes can alter the electrodeposition process by influencing their electrochemical window.^41^

**Figure 4:**
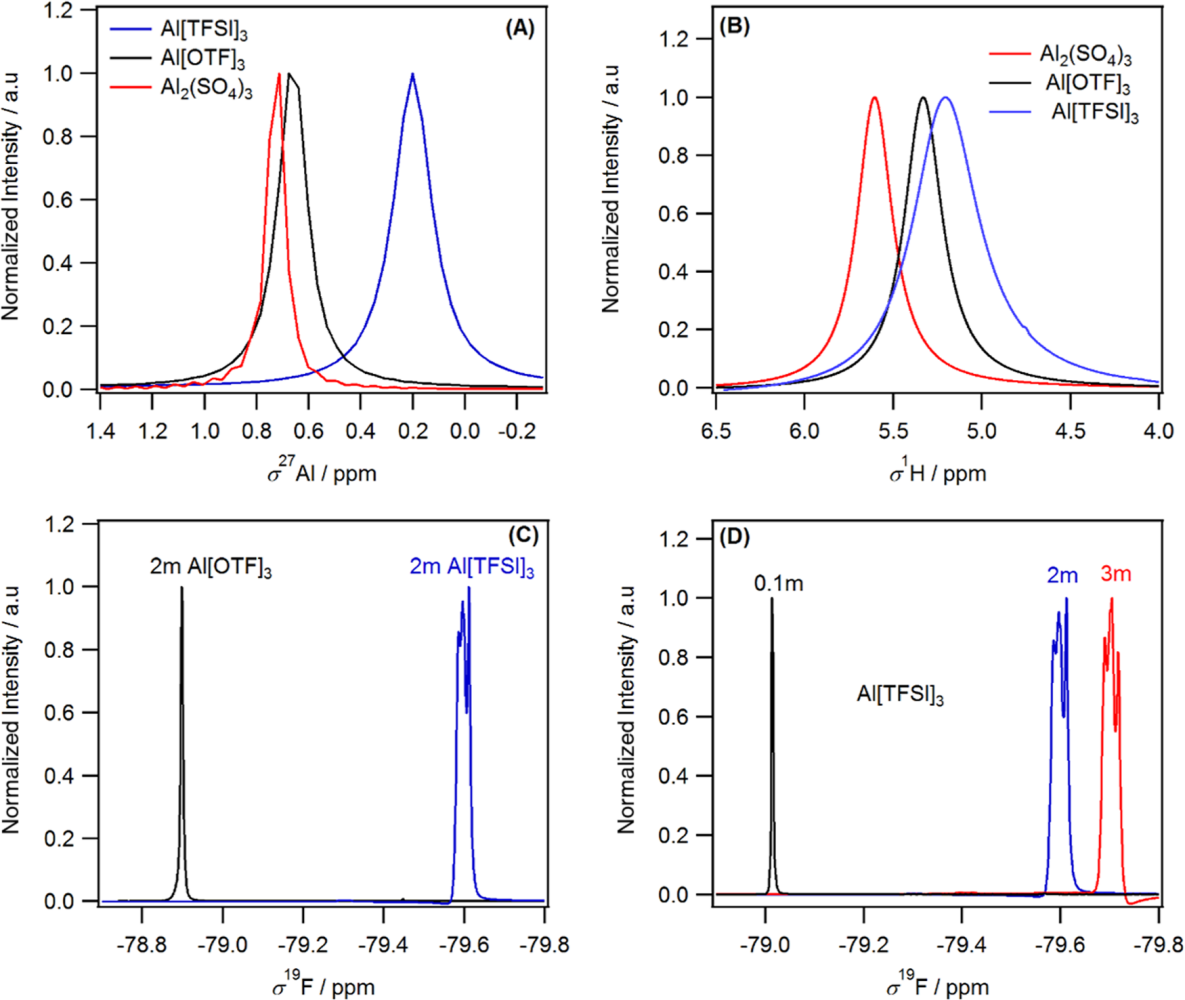
Normalized NMR spectra of aqueous Al electrolytes showing (A) ^27^Al spectra of 3m Al[TFSI]_3_, 2m Al[OTF]_3_, and 2m Al_2_(SO_4_)_3_; (B) ^1^H spectra of 3m Al[TFSI]_3_, 2m Al[OTF]_3_, and 2m Al_2_(SO_4_)_3_; and (C) and (D) ^19^F spectra using the concentration given in the figure panels.

The solvation structure of the three Al electrolytes was explored using ^27^Al, ^1^H, and ^19^F NMR and other spectroscopic techniques. Figure 4A shows that the signal due to ^27^Al moved to a lower chemical shift as the Al electrolyte counteranion changed from SO_4_^2–^ (0.71 ppm), [OTF]^−^ (0.65 ppm) to [TFSI]^−^ (0.2 ppm). This suggests that the Al cation in Al[TFSI]_3_ is more shielded than the Al^3+^ cation in Al[OTF]_3_ or Al_2_(SO_4_)_3_ electrolyte. The increase in electronic density around Al^3+^ in [TFSI]-based electrolyte indicates a change in the solvation shell of Al^3+^, perhaps through the formation of ionic aggregates (TFSI-TFSI) and contact ion pairs (TFSI-Al^3+^).^29^ A decrease in the chemical shift of ^27^Al was also observed when the concentration of Al[TFSI]_3_ increased, which results in an increase in the electronic density around Al^3+^ (Figure S7); presumably, water (or hydroxide) is being displaced from the coordination environment of the Al and replaced with the [TFSI] anion. This observation is consistent with the NMR of LiTFSI electrolytes.^29^ Similarly, a larger decrease in the chemical shift of ^1^H and ^19^F was observed for the [TFSI]^−^ anion compared to that of [OTF]^−^ or SO_4_^2–^ anions, and the extent of shielding increased with increasing [TFSI]^−^ concentration (Figure 4B,D). Fourier transform infrared (FTIR) spectroscopy (Figure S8) also showed that the characteristic water (O–H) stretching mode shifted to higher wavenumbers for Al[TFSI]_3_ when compared to that for Al[OTF]_3_ or Al_2_(SO_4_)_3_. In addition, the NMR spectrum due to ^19^F showed a coupling pattern with increasing Al[TFSI]_3_ concentration. The coupling could be with the proton from water or through close Al^3+^–anion interactions, but the fact that the coupling occurs when the concentration of Al[TFSI]_3_ increased suggests that the contact ion pair (TFSI-Al^3+^) interaction might be the cause.^29^ Alternatively, it could be due to the coupling of anion–anion interaction through aggregates of the [TFSI] network. A recent study of [TFSI] solvation structure using synchrotron X-ray scattering showed two coexisting structures: [TFSI]-solvated structure (caused by hydrogen bonding between the bulk water and [TFSI]^−^) and [TFSI] network (formed through the hydrogen bonding between the interfacial water and [TFSI]^−^) depending on its concentration. As the concentration of [TFSI]^−^ anions increases, even at relatively low concentration, the [TFSI] network gradually forms, while the [TFSI] solvated structure gradually disappears.^42^ The formation of [TFSI] aggregates significantly alters the solvation structure of the electrolyte and is responsible for the increase in the electrochemical window of the electrolyte.^42^ Overall, spectroscopic data analysis indicated that the solvation structure of Al^3+^ in [TFSI] anions is considerably different from the other two anions and this is responsible for the difference in electrochemical performance.

**Figure 5:**
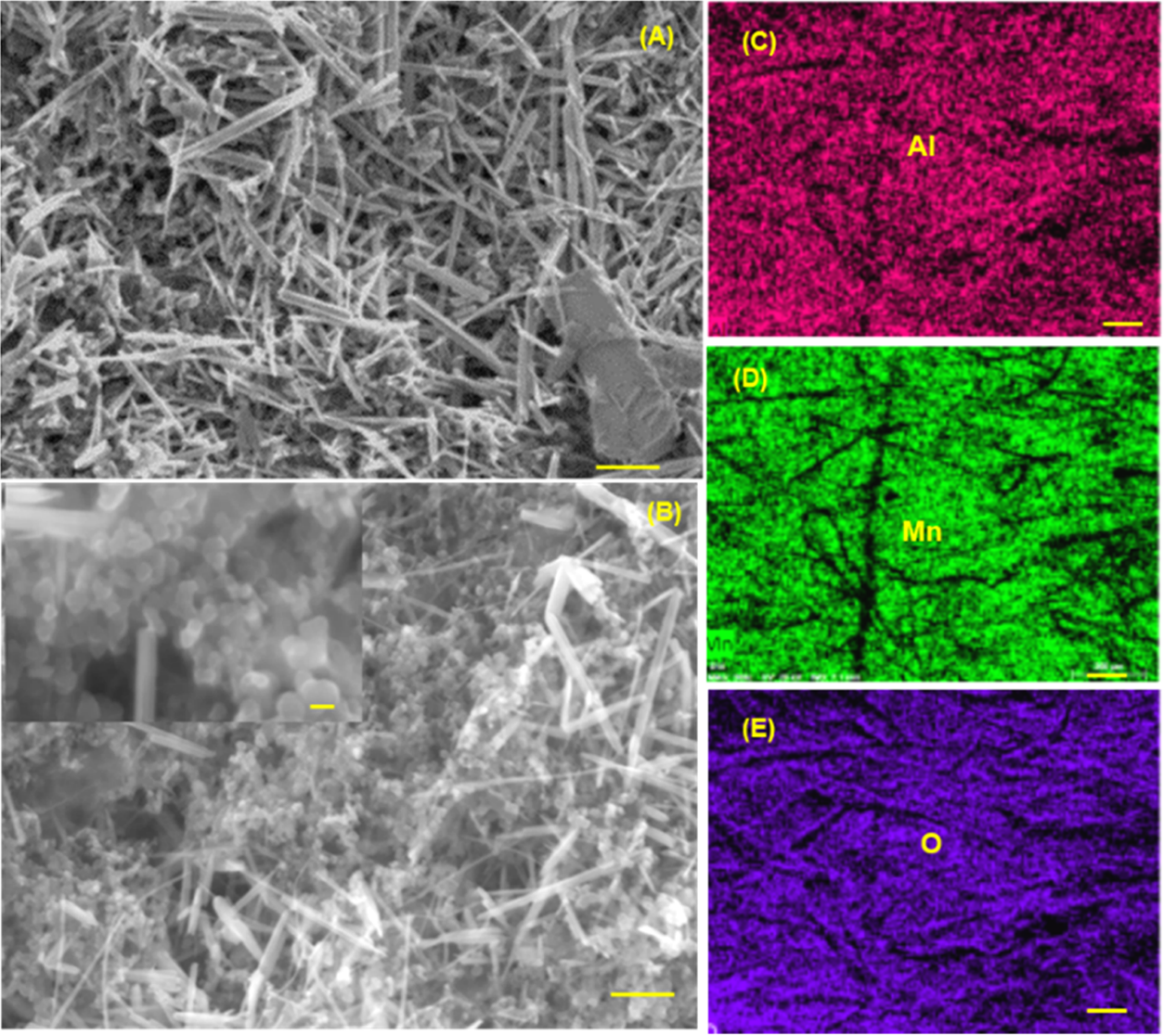
SEM images of (A) pristine MnO_2_ (scale bar is 1 μm) and (B) discharged MnO_2_ electrode in 3m Al[TFSI]_3_; the inset shows the high magnification. The scale bar is 1 μm and the inset is 0.2 μm. Panels (C–E) show the elemental mapping of the discharged MnO_2_ electrode at shown elements. The scale bar in each case is 100 μm.

A combination of electron microscopy, XRD, and spectroscopic analysis was also carried out to get insights into the electrochemical processes at the positive electrode. The morphology of the fully discharged MnO_2_ electrode in Al[TFSI]_3_ was analyzed and compared to pristine MnO_2_ after washing several times with water to remove the residual electrolyte. The SEM image of pristine MnO_2_ exhibited rodlike nanostructures with an average length of ∼2 μm (Figure 5A). The morphology of the MnO_2_ electrode significantly changed when the cell was fully discharged. SEM images showed the presence of smaller nanostructures that range between 100 and 500 nm in size (Figure 5B), presumably due to the insertion of guest species into MnO_2_ (this will be discussed in the next section). EDX mapping revealed the homogeneous distribution of the Al element on the MnO_2_ electrode (Figure 5C). A trace amount of Zn^2+^ was also inserted alongside the Al^3+^ into the MnO_2_, with the atomic ratio of Zn/Mn being 0.05. The trace Zn^2+^ obtained at the positive electrode as well as in the electrolyte is most likely due to the crossover of some of the oxidized Zn^2+^ during cell discharge, as also seen previously.^16^ However, it is important to note that Zn^2+^ does not seem to contribute to the capacity of the cathode given that Zn^2+^ chemistry at the MnO_2_ cathode usually occurs at <1.4 V (Figure S9).

**Figure 6:**
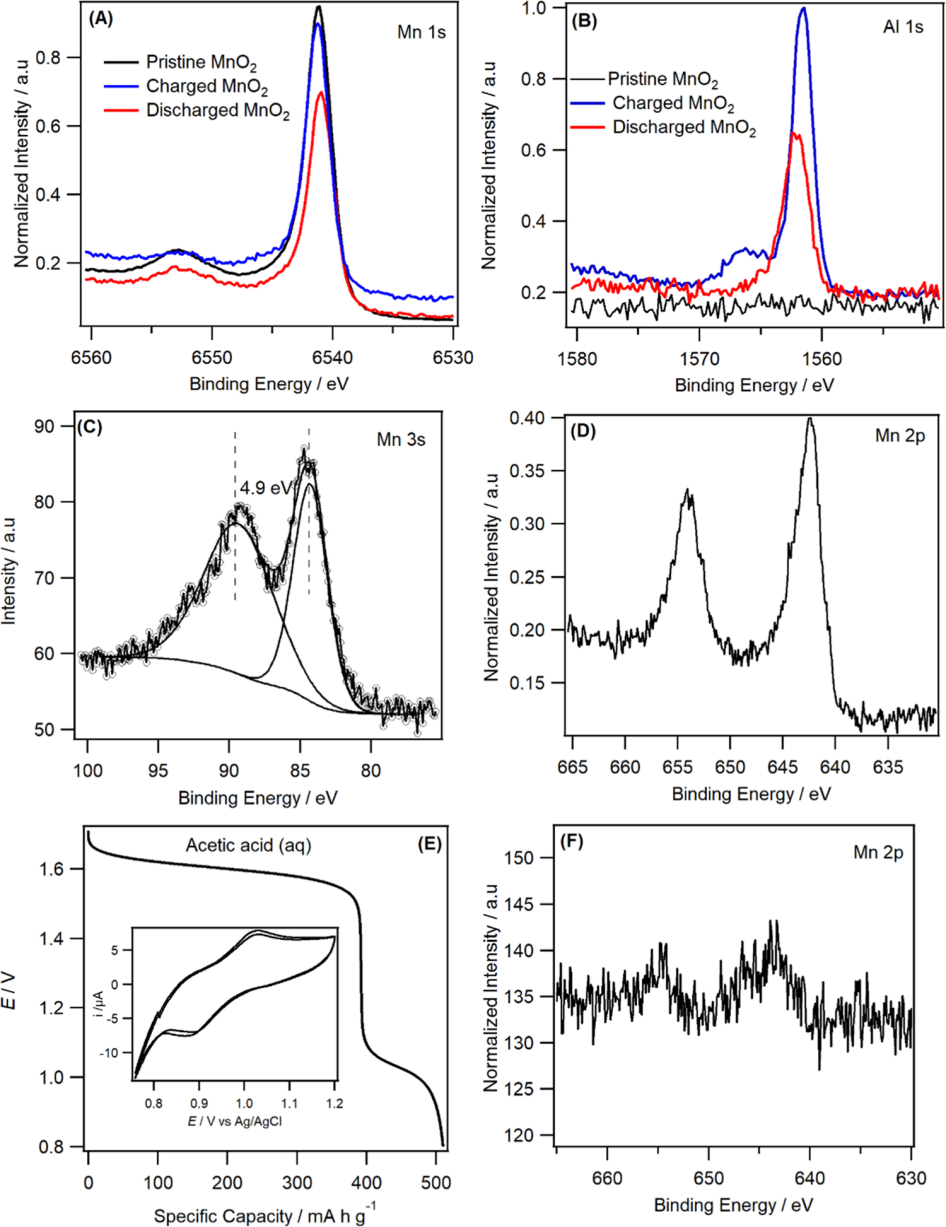
High-resolution HAXPES spectra of charge–discharged MnO_2_ cell in 3m Al[TFSI]_3_ in the region of (A) Mn 1s, (B) Al 1s, (C) Mn 3s discharged, and (D) Mn 2p discharged MnO_2_. (E) Discharge curve obtained using aqueous acetic acid solution (p^H^ =1.2) at Zn–Al*_X_*/acetic acid (aq)/MnO_2_ cell at an applied current of 75 mA g^–1^. The inset shows the CVs obtained at a MnO_2_ working electrode in acetic acid solution using Pt and Ag|AgCl as counter and reference electrodes, respectively. (F) High-resolution HAXPES spectra of the fully discharged MnO_2_ electrode from acetic acid (aq) solution in the Mn 2p region.

*Ex situ* hard X-ray photoelectron spectroscopy (HAXPES, 9.25 keV) and conventional XPS (1.486 keV) were used to examine the change in the manganese oxidation state during the cell charge–discharge process (Figures 6 and S10). The sampling depth of MnO_2_ based on Mn 2p for HAXPES is 34.1 nm compared to only 5.3 nm for XPS.^43,44^ HAXPES is able to probe below the topmost surface layer and thus can be used to infer information from the material surface toward the bulk.^44^ Comparing information extracted using the two photon energies therefore enables the assessment of any changes occurring at the surface to subsurface. In addition, HAXPES also enables the use of deeper core levels at relatively higher binding energies, such as Mn 1s (∼6540 eV BE) and Al 1s (∼1560 eV). The Mn 1s core level is useful because no multiplet splitting effects are expected; the spectra for the fully discharged, charged, and pristine MnO_2_ all show one symmetric peak at 6541.0, 6541.2, and 6541.1 eV, respectively (Figure 6A). The lack of difference in binding energy indicates that Mn is present as MnO_2_ for charge–discharged samples. In addition, Al 1s is present at 1562.5 eV (metallic Al exhibits Al 1s at ∼1559.8 eV^45^) for the discharged electrode, where the positive binding energy shift compared to the metallic state confirms the insertion of Al^3+^ (Figure 6B). In the charged MnO_2_, an additional peak at a higher binding energy of 1566.0 eV is seen, which could be due to the formation of aluminum oxide.

Measurement of the Mn 3s core level is also useful to determine the oxidation state of Mn. Mn 3s splits due to multiplet splitting effects caused by unpaired d-electrons, and the magnitude of the splitting can reveal the oxidation state of Mn.^46^ HAXPES measurements obtain a 3s core level splitting width of ∼4.9 eV for both charged and discharge samples, suggesting that the oxidation state of Mn remains unchanged.^47,48^ The XPS spectra for Mn 3s for charge–discharged electrodes, however, are perturbed by additional peaks in this region originating from Al KLL (Auger transition) and trace Zn (Zn 3p), which overlap, as shown in Figure S10C. This makes use of the 3s core level challenging when using XPS. The lack of this structure in HAXPES suggests that Zn is mainly present at the surface as impurities, while the Al Auger transition has been shifted to a different binding energy position as the photon energy has changed. The fact that Zn is absent below the subsurface suggests that Zn does not contribute to the capacity of the cathode. For the discharged and charged cells, the Mn 2p_3/2_ binding energy positions were 642.3 and 642.5 eV using XPS and 642.4 and 642.6 eV using HAXPES. This suggests that the Mn is present as MnO_2_ in both samples at the surface as well as in the bulk, i.e., similar HAXPES and XPS binding energy positions may indicate a homogeneous chemical state.^49^ Note that we only take the peak position of Mn 2p_3/2_; peak fitting of the first two transition-metal 2p core levels is challenging due to multiplet splitting effects caused by unpaired d-electrons [7]. The HAXPES and XPS data indicate that there is little or no change from the surface into the bulk of the material when the MnO_2_ electrodes undergo charge–discharge process.

**Figure 7:**
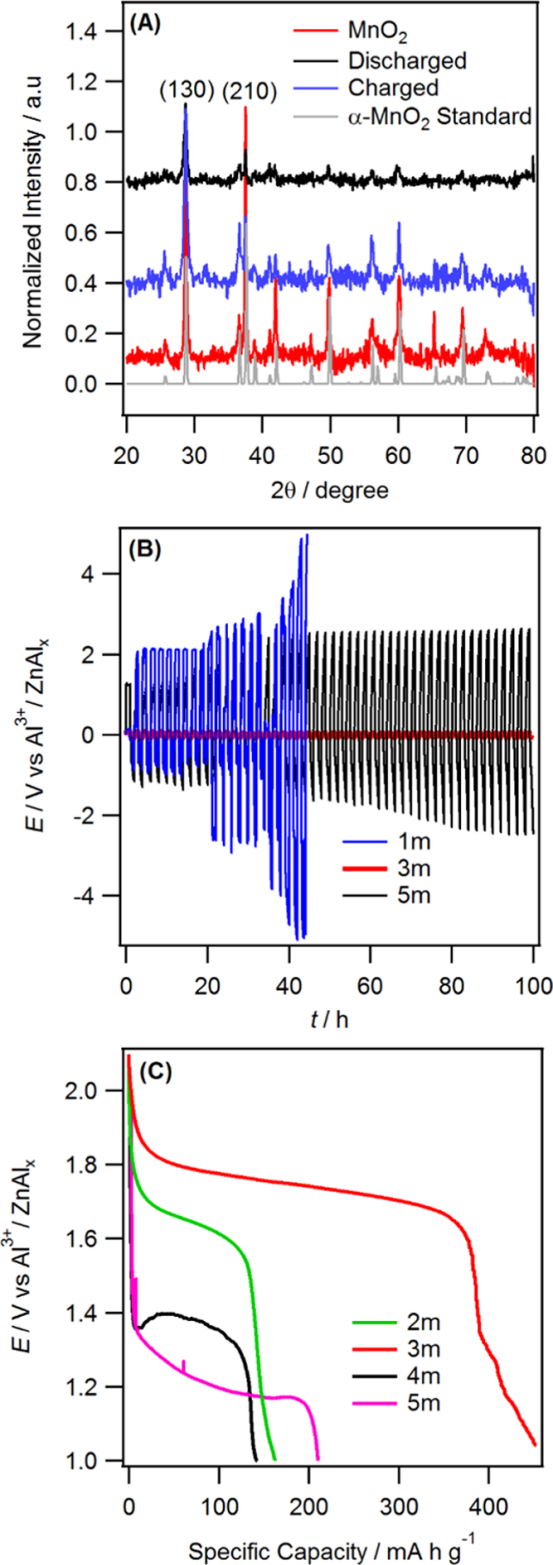
(A) XRD patterns of MnO_2_ cathodes at fully discharged and charged states, (B) galvanostatic charge–discharge curve obtained using the symmetrical Zn|Zn cell in 1.0, 3.0, and 5.0m Al[TFSI]_3_ electrolytes at 0.2 mA cm^–2^, (C) galvanostatic charge–discharge curves vs capacity obtained at a current density of 25 mA g^–1^ using various Al[TFSI]_3_ electrolytes.

Given that both HAXPES and XPS showed that the oxidation state of Mn remains unchanged during the battery charge–discharge mechanism, what is the active redox species at the cathode then? There are controversies in the literature regarding the active species (Al^3+^ or H^+^) intercalated into MnO_2_ during battery discharge. Previous work suggested that the insertion of Al^3+^ into the oxide is accompanied by the cointercalation of water, which form a layered Al*_x_*MnO_2_·*n*H_2_O phase.^37^ It was believed that this coinserted water crystal is critically important for the reversible intercalation of Al^3+^ into the oxides by insulating the electrostatic interaction between the Al^3+^ ion and the host MnO_2_.^37,50^ A similar mechanism was suggested using other multivalent ions including Mg^2+^ and Zn^2+^ during the electrochemical cycling of manganese oxide electrode in aqueous electrolytes.^50,51^ However, recent research has questioned whether Al^3+^ intercalation into MnO_2_ occurs at all and, instead, proposed that proton insertion is the dominant reaction mechanism. Wang et al. studied the charge storage mechanism of α-MnO_2_ in the Al[OTF]_3_ electrolyte using various electrochemical/spectroscopic characterizations and found that proton intercalation/deintercalation largely contributes to the reversible capacity of the cell, while only a small amount of Al^3+^ could also intercalate into MnO_2_.^52^ It also proposed that a complex surface product containing Al^3+^, OH^–^, and [OTF]^−^ was formed during cell discharge and this product dissolves during the charging process. Alternatively, Balland et al. used an *in situ* spectroelectrochemical methodology to determine the reaction mechanism of Al^3+^ in the MnO_2_ electrode using the Al[OTF]_3_ electrolyte.^53^ They proposed a mechanism based on the reversible proton-coupled MnO_2_ to Mn^2+^ conversion where the hydrated Al^3+^ acts as a proton donor.^53^ We also investigated the insertion of proton using CV and galvanic charge–discharge curves employing an aqueous acetic acid electrolyte with a similar pH (1.2) value to the 3m Al[TFSI]_3_ electrolyte. The cell was discharged from the open circuit potential (OCP) value. The interesting points observed were as follows: first, the OCP of the cell was 1.72 V, similar to the Al[TFSI]_3_-containing electrolyte. Second, when the cell was fully discharged, the battery displayed a similar discharge plateau to the Al[TFSI]_3_ electrolyte but with a much enhanced discharge capacity of 510 mAh g^–1^ (see Figure 6E). This capacity is close to the theoretical value for the conversion of the Mn^4+^/Mn^2+^ reaction.^54^ In addition, CVs showed the reversible insertion of proton into the MnO_2_ electrode (inset of Figure 6E). To get further insight, we analyzed the discharged MnO_2_ electrode in aqueous acetic acid solution using HAXPES. We observed that only a trace amount of manganese species detected with an atomic concertation of Mn is 0.3%. This demonstrates that the majority of Mn^4+^(MnO_2_) was reduced to Mn^2+^, which then dissolves away from the electrode, confirming that proton insertion reduces Mn^4+^ to Mn^2+^ redox reaction. The fact that we observed the presence of Mn species along with Al, during HAXPES analysis for Al[TFSI]_3_, indicates that the intercalation of proton was not the sole reaction mechanism. Instead, a small amount of Al^3+^ also cointercalates into MnO_2_ with the atomic ratio of Al to Mn being 0.07 according to HAXPES for the fully discharged sample. This was, however, small enough to alter the oxidation state of Mn to be detected by HAXPES (Al_0.07_MnO_2_). Given that the theoretical capacity of Al_0.07_MnO_2_ is only 65 mAh g^–1^, the dominant reaction mechanism occurring at the MnO_2_ electrode when using Al[TFSI]_3_ electrolyte is proton insertion donated from hydrated Al^3+^ (eq 1) rather than Al^3+^ insertion (eq 2), which is consistent with the recent work.^52,53^ The MnO_2_ positive electrode structural reversibility after the charge–discharge process was also examined using *ex situ* XRD. Figure 7A shows the XRD pattern of pristine MnO_2_, which was well indexed to the α-MnO_2_ phase. The prominent (130) and (210) peak intensities of MnO_2_ are weakened, while the other peaks disappeared when the cell was fully discharged. All of the diffraction patterns are recovered when the cell was charged, indicating the structural reversibility of MnO_2_
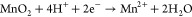
1
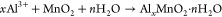
2

### 2.3 Effect of Al[TFSI]_3_ Concentration on Cell Performance and Stability

Given that the performance of Al[TFSI]_3_ is superior to that of Al[OTF]_3_ or Al_2_(SO_4_)_3_ when used in AIBs, the effect of varying Al[TFSI]_3_ concentrations on the cell performance is further optimized. We prepared Al[TFSI]_3_ solutions with varying salt concentrations, and the maximum limit of the Al[TFSI]_3_ solubility in water was found to be 5m. The concentration of electrolytes has a profound effect on the chemistries and cell performance of several batteries including Li-ion, Al-ion, and Zn-ion batteries.^16,18,55^ It has also been suggested that the electrolyte properties are largely affected by the identity of the anion, rather than the cation, during aqueous electrolyte formulation.^21^ In this case, not only the concentration or the nature of anions impacts but also the stoichiometry between the cation and anion should be considered. For example, one may get the same number of [TFSI] ions in a solution of 9m LiTFSI and 3m Al[TFSI]_3_, with a relatively low concentration of Al[TFSI]_3_; the water-in-salt regime may be achieved. The kinetics of Al^3+^/Al deposition and stripping of the different Al[TFSI]_3_ salt concentrations were evaluated in Zn|Zn symmetric cells. Figure 7B displays the typical voltage–time profiles at a constant charge and discharge current density of 0.2 mA cm^–2^. The charge–discharge profile in the 1m Al[TFSI]_3_ electrolyte displayed a large overpotential for the Al^3+^/Al reaction that was increased with cycling until the cell failed within 50 h. This could be due to the formation of water-induced irreversible side reactions including hydrogen evolution and associated surface oxidation. For the 5m Al[TFSI]_3_ (as well as for the 4m concentration) electrolyte, the symmetric cell also exhibited a large polarization potential although cell failure was not observed. There are a few factors that may contribute to the slow electrode kinetics of Al^3+^/Al in 5m Al[TFSI]_3_. The ionic conductivity measurement using AC impedance showed that conductivity decreased from 46.9 mS cm^–1^ in 3m Al[TFSI]_3_ to 4.8 mS cm^–1^ in 5m Al[TFSI]_3_. The significant conductivity decrease (caused by the associated viscosity increase) may affect the rate of Al^3+^/Al reaction as well as the diffusion of Al^3+^. In addition, the surface morphology of the Al electrodeposits obtained using 5m Al[TFSI]_3_ considerably differs from the 3m, as shown in Figure S11. The data and discussion of further electrolyte characterization using Fourier transform infrared spectroscopy, UV–vis absorption spectroscopy, and Raman Spectroscopy are presented in the Supporting Information (SI). Excellent reversibility of Al stripping/plating with the smallest polarization was achieved in the 3m Al[TFSI]_3_ electrolytes. The reversibility of Al^3+^ chemistry was also compared using 5m and 3m Al[TFSI]_3_ salt concentrations as an example. As shown in Figure S12A, the peak-to-peak separation for Al^3+^/H^+^ insertion and extraction significantly increased from 0.4 V in 3m to 0.75 V in the 5m Al[TFSI]_3_ electrolyte. In addition, the current measured was significantly decreased for the 5m electrolyte, which is most likely due to the slow rate of Al^3+^/Al transport caused by the associated viscosity increase. The AIB cell with the 3m Al[TFSI]_3_ exhibits the best electrochemical performance. It shows a specific discharge capacity of 450 mA h g^–1^ at a current of 25 mA g^–1^ compared to that of <250 mA h g^–1^ for the other electrolytes. In addition, the discharge voltage plateau and CE decreased as the concentration of Al[TFSI]_3_ increased from 3m to 5m, mirroring the slower kinetics of Al^3+^/Al at concentrations >3m. For example, the CE of the 3m system is ∼90% compared to that of 74% at 5m. This data highlights that 3m Al[TFSI]_3_ is the optimum electrolyte concentration for use in AIBs.

**Figure 8:**
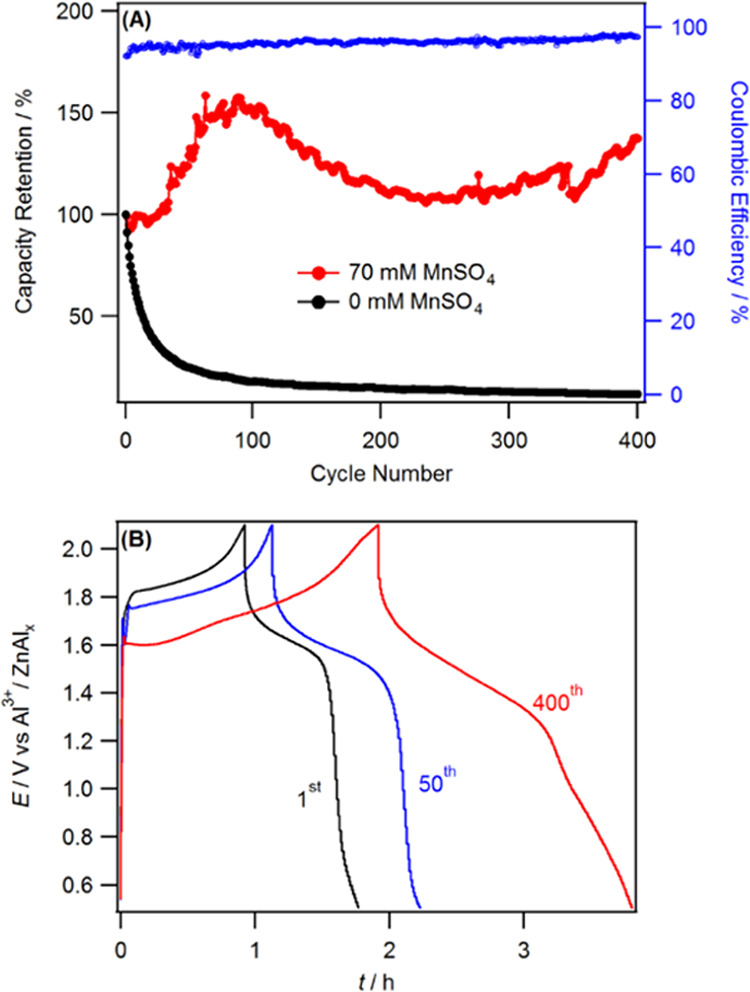
(A) Capacity retention and Coulombic efficiency of Zn–Al*_X_*|MnO_2_ cell cycled at 0.15 A g^–1^ using 3m Al[TFSI]_3_ electrolyte and (B) charge–discharge curve obtained at 0.15 A g^–1^ at the shown cycling stage.

Finally, the stability of the AIBs cell was examined: Figure 8A shows the capacity retention of Zn–Al_*X*_|3m Al[TFSI]_3_|MnO_2_ as a function of cycling number. The capacity of the cell decayed rapidly to less than 20% within the first 20 cycles in the electrolyte where there is no Mn^2+^ additive present. Previous research on the use of MnO_2_ cathodes in both Zn-ion cells and AIBs showed that MnO_2_ suffers a structural loss due to the dissolution of Mn^2+^ during cell discharge.^15,56,57^ This Mn^2+^ leaching is generally responsible for the rapid capacity fade during cell operation. Preaddition of Mn^2+^ salt is widely considered the most effective strategy to enhance capacity retention although the mechanism behind this remains controversial.^58,59^ Indeed, the cell that contained the Al[TFSI]_3_ electrolyte with Mn^2+^ additives exhibited excellent cyclic stability throughout the 400 cycles. The capacity initially increased by 50% during the first 100 cycles and then stabilized at a little more than the initial value. The increase in capacity could be due to the gradual activation of the surface area by the electrodeposition process of the Mn^2+^ additives. Examination of the charge–discharge curve showed that the average voltage plateau of the cell during the charging process progressively decreased, while the length of the discharging curve increased with an increase in cycle numbers (Figure 8B). This indicates that the overall rate of H^+^/Al^3+^ insertion/extraction from the positive electrode and Al plating/stripping at the negative electrode are enhanced with cycling. In addition, the CE increased from 94 to 99% with increased cycling. When compared to the Al[TFSI]_3_ electrolyte, [SO_4_]^2–^- or [TFO]^−^-based electrolytes did not exhibit good cycling stability even with Mn^2+^ additives. This was due to electrolyte degradation to form hydrogen evolution, as evidenced by the low CE (25% in Al_2_(SO_4_)_3_ compared to over 95% in Al[TFSI]_3_) (Figure S13). We also note that the 3m Al[TFSI]_3_ electrolyte performed well with the spinel-structured Mn_3_O_4_ cathode. As shown in Figure S14, the electrochemical behavior (CV) and the battery performance of Mn_3_O_4_ are comparable to the α-MnO_2_ cathode. We believe that these findings may promote the development of energy-dense aqueous AIBs.

## 3 Conclusions

The development of novel aqueous aluminum-based electrolytes with the ability to suppress the hydrogen evolution side reaction, and which overcome the issue of Al passivation, is crucially important for the realization of aluminum-ion batteries. We investigated the suitability of three different Al electrolytes in terms of their practicality for aqueous AIBs. Our results showed that the nature of the electrolyte anion has a crucial effect on the electrochemical window of water, the surface morphology of aluminum electrodeposits, the degree of Al^3+^/Al reversibility, and the performance of the cell. An Al electrolyte based on bis(trifluoromethanesulfonyl)imide anion suppressed the hydrogen evolution side reactions and enabled the cell operating window to extend to 2.1 V. The accessibility of the high-voltage window by the Al[TFSI]_3_ allowed the reversible electrochemistry Al^3+^ at the anode, thereby achieving a record discharge voltage of 1.75 V with a high capacity of 450 mAh g^–1^. In addition, the use of this innovative electrolyte produced Zn–Al alloy electrodeposits that consist of uniform nanostructures that are less prone to dendrite formation with excellent reversibility for Al electrochemistry when compared to the electrodeposits obtained from Al[OTF]_3_ or Al_2_(SO_4_)_3_ electrolyte. The results of this study can be seen as a proof of concept for further Al electrolyte development. In particular, solid electrolyte interface forming anions based on various perfluorinated sulfonylimides and their mixtures should be investigated to further fine-tune the performance of AIBs with an understanding of their interfacial properties. In addition, it is also important to consider the costs when evaluating the feasibility of using [TFSI]-based electrolytes in aqueous batteries. While [TFSI]-based salts remain relatively expensive, such prices will fall as the salt’s popularity grows.

## 4 Experimental Methods

### 4.1 Materials and Apparatus

Anhydrous aluminum chloride (99.99%), aluminum trifluoromethanesulfonate (99.9%), aluminum sulfate (99.99%), KMnO_4_ (99%), MnSO_4_·H_2_O, and *N*-methyl-2-pyrrolidone were obtained from Sigma-Aldrich and used as received. Titanium (99.99%) and aluminum foil (99.99%) were obtained from Alfa Aesar. Trifluoromethanesulfonimide (95%) was obtained from Fluorochem. X-ray photoelectron spectroscopy (XPS) was performed using a Kratos Axis Ultra DLD spectrometer with a monochromated Al Kα X-ray source (*E* = 1486.6 eV, 10 mA emission). Hard X-ray photoelectron spectroscopy (HAXPES) was performed using the monochromated Ga Kα X-ray radiation (9250 eV, 3.0 mA emission at 210 W, microfocused to 50 μm) and an EW-4000 high-voltage electron energy analyzer (HAXPES-Lab, Scienta Omicron GmbH); the instrument has a base vacuum pressure of ∼5 × 10^–10^ mbar.^44,60^ The pass energies used for survey and core level spectra were 500 and 200 eV, respectively, using analyzer entrance slit widths of 1.5 and 0.8 mm, respectively, with total energy resolutions of 2.0 and 0.6 eV, respectively, as measured using the FWHM of the Au 4f_7/2_ core level on a clean gold reference sample. The HAXPES instrument is also equipped with a monochromated Al Kα X-ray source (1486 eV, 20 mA emission at 300 W) for surface-sensitive XPS at the same sample position (although with a larger spot size of ∼1 mm diameter). Survey and core level spectra were measured using 200 and 50 eV pass energies, respectively (using a 1.5 mm wide entrance slit), with a total energy resolution approximately 50% compared to HAXPES. Binding energy scale calibration was performed using C–C in the C 1s photoelectron peak at 284.8 eV. Analysis and curve fitting were performed using Voigt-approximation peaks using CasaXPS [3]. Core level relative sensitivity factors for HAXPES quantification were calculated according to ref (44). Al^27^ NMR data were collected using a Bruker Avance II+ 500 MHz NMR spectrometer equipped with a 5 mm BBI probe. ^1^H and ^19^F data were collected using a Bruker Avance III 400 MHz NMR spectrometer equipped with a 5 mm Bruker Prodigy Cryo probe. All NMR data were collected at 298 K. Raman spectra were obtained using a Renishaw inVia microscope with a 532 nm excitation laser operated at a power of 0.274 mW with a grating of 1800 lines/mm and 50× objective. SEM analysis was carried out using an FEI Quanta 650 FEG environmental scanning electron microscope. Powder X-ray diffraction analysis was performed using a Philips X’pert PRO diffractometer with Cu Kα radiation (λ = 0.154 nm) and operating at 40 kV and 30 mA. UV–vis spectroscopy was measured using a model DH-2000-BAL (Ocean Optics). Electrochemical measurements were performed using an Autolab potentiostat (model PGSTAT302N, Metrohm Autolab, The Netherlands). The charge–discharge battery test was carried out using a Basytec cell test system (GmbH, Germany) with 32 independent test channels.

### 4.2 Preparation of Aluminum Bis(trifluoromethanesulfonyl)imide

Aluminum bis(trifluoromethanesulfonyl)imide (Al[TFSI]_3_) was synthesized according to the experimental procedure reported in the literature following eq 1.^61^ In brief, trifluoromethanesulfonimide was fully melted by heating to 60 °C followed by the slow addition of AlCl_3_ (1.05:1.0 molar ratio of acid to AlCl_3_, respectively) under an argon-filled glovebox. The mixture was stirred at 60 °C for 12 h. The HCl byproduct was removed by heating under a vacuum oven overnight at 120 °C. It is preferable to use the Schlenk line to avoid damage to the vacuum oven by HCl. The complete reaction and removal of the HCl were confirmed by X-ray photoelectron spectroscopy and NMR spectroscopy. Each of the Al salt solutions was prepared using ultrapure water, and to aid the dissolution of Al[TFSI]_3_ in water, the mixture was heated to 70–80 °C while stirring. The ionic conductivity of the solutions was determined by AC impedance spectroscopy in a U-shaped glass cell that was filled with the desired electrolytes and contained two identical Ag wires held at a fixed distance from each other. The cell constant was determined using a 0.0145 M KCl conductivity standard (Alfa Aesar). The solution resistance (*R*_S_) was obtained from the horizontal intercept of the Nyquist plot in the high-frequency region and used for ionic conductivity calculation
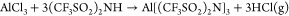
3

### 4.3 Preparation of α-MnO_2_ Nanorods

α-MnO_2_ was prepared by a traditional hydrothermal method.^62^ Briefly, 0.50 g of KMnO_4_ and 0.21 g of MnSO_4_·H_2_O were added to 32 mL of deionized water to form a homogeneous solution under magnetic stirring for about 10 min. The solution was then transferred to a Teflon-lined stainless steel autoclave and heated in an oven at 160 °C for 12 h. The obtained product was collected by filtration, washed with deionized water, and dried at 80 °C in an oven overnight.

### 4.4 Electrode Preparation

The α-MnO_2_ slurries were prepared using 0.8 g of α-MnO_2_, 0.1 g of carbon black/Super P, and 0.1 g of poly(vinylidene fluoride) (Kynar 761 PVDF) binder in a sufficient volume of *N*-methyl-2-pyrrolidone (NMP). The slurry was stirred overnight and coated over a Ti (99.95%) substrate or nonwoven carbon (Technical Fibre Products, U.K.) substrate using doctor blading. The resulting cast electrodes were dried in a vacuum at 80 °C overnight. The cast electrodes were then punched into small disks of 1 cm diameter for use in a coin cell. The mass loading of the electrodes ranged from 2 to 5 mg cm^–2^. The Zn–Al*_X_* anode electrodeposits were prepared in situ by charging the cell from the cell open circuit voltage at a rate of 25 mA g^–1^ using a Zn foil anode, an α-MnO_2_ cathode, and an Al-based aqueous electrolyte.

### 4.5 Battery Assembly and Electrochemical Measurements

The full cells were assembled in CR2032-type coin cells using α-MnO_2_ as the cathode, Zn foil as the anode, a glass microfiber filter as the separator and 150 μL electrolytes. The electrolytes were made by dissolving various Al salts (Al[TFSI]_3_, Al[OTF]_3_, or Al_2_(SO_4_)_3_) with various concentrations in ultrapure water (Millipore Milli-Q), which was previously purged with Ar gas for 30 min. Coin cells were assembled using a hydraulic crimping machine (MSK-160D) in an ambient atmosphere. For Coulombic efficiency (CE) determination for Al deposition and stripping at the Zn electrode, an asymmetric cell consisting of Zn and carbon electrodes in addition to the Al[TFSI]_3_ electrolyte was used. The cell was set to electrodeposit the alloy for 30 min at 2 mA cm^2^ and follow the CE by stripping the electrodeposited Al. Three-electrode cell electrochemical measurements were conducted using a 3 mm diameter glassy carbon (GC) disk as the working electrode, a Pt wire as the counter electrode, and Ag|AgCl as the reference electrode. The electrodeposition of Zn on GC was carried out using 0.1m ZnCl_2_(aq), and the GC/Zn electrode was used as the working electrode for the electrochemistry of Al electrodeposition study from the Al[TFSI]_3_ electrolyte (Figure S2A).

## Supporting Information Available

NMR spectroscopy of various elements, XPS, cyclic voltammograms, and galvanostatic charge–discharge curves of various electrodes, Raman and FITR spectroscopy, and SEM (PDF)
