# Supplementary material for: Optimization of Electrolytes for High-Performance Aqueous Aluminum-Ion Batteries
Source: ACS Appl Mater Interfaces. 2022 May 27;14(22):25232–45. doi: 10.1021/acsami.1c23278 (PMC9185688; doi:10.1021/acsami.1c23278)
Supplement: Supplementary file 1 — am1c23278_si_001.pdf [file am1c23278_si_001.pdf]

Supporting Information
Optimization of Electrolytes for High Performance Aqueous Aluminum ion Battery

Andinet Ejigu[a,b*], Lewis W. Le Fevre[b], Amr Elgendy[a,b], Ben F. Spencer[c], Carlo Bawn[a] and Robert A.W. Dryfe[a,b*]

[a]Dept. of Chemistry, University of Manchester, Oxford Road, Manchester M13 9PL, UK

[b]Henry Royce Institute, University of Manchester, Oxford Road, Manchester, M13 9PL, UK

[c] Dept. Of Materials, University of Manchester, Oxford Road, Manchester, M13 9PL, UK

Corresponding Authors:

E-mail: andinet.aynalem@manchester.ac.uk

E-mail: Robert.dryfe@manchester.ac.uk

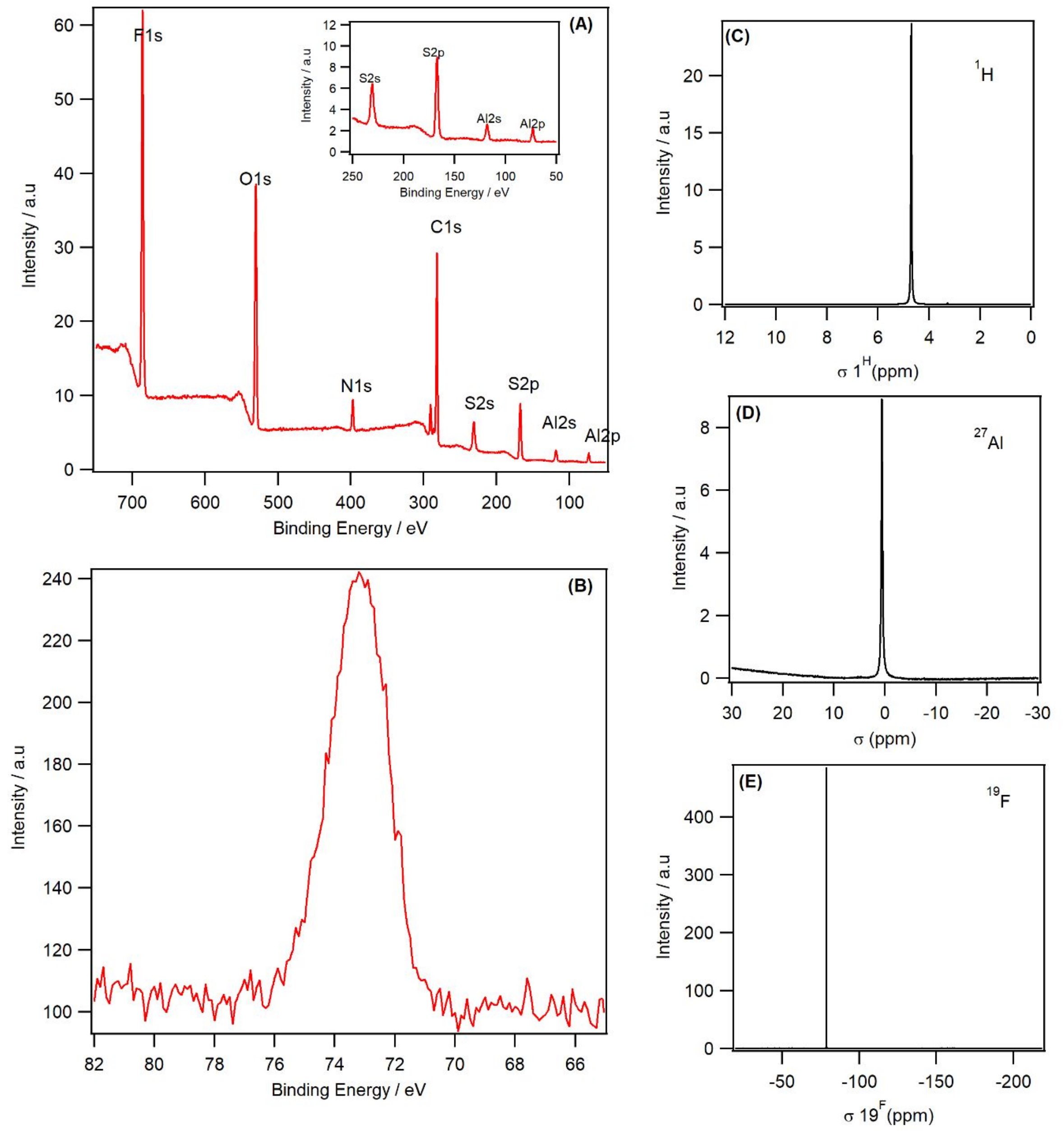

Figure S1 *A) Survey scan XP spectrum of neat Al[TFSI]$_3$, (B) high resolution XP spectra in the Al2p region. NMR spectroscopy: (C)^{1}HNMR of Al[TFSI]$_3$ (showing the absence of the acidic proton which would have seen around 9-10 ppm;the spectrum at 4.8 ppm is due to D_2O reference solvent) and (D)^{27}Al NMR and (E)^{19}F NMR of Al[TFSI]$_3$*

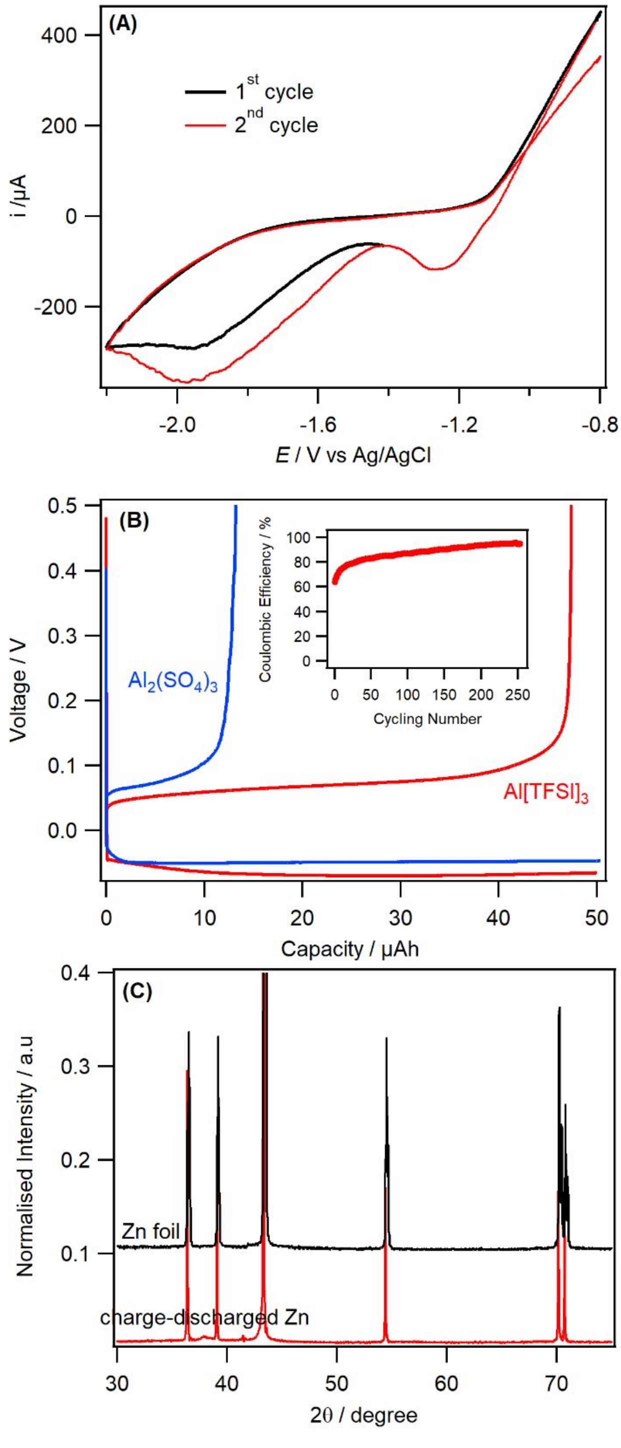

Figure S2. *(A) CVs recorded at Zn electrode (electrodeposited on 3mm diameter disk GC electrode) in three-electrode configuration at 5 mV s^{-1} using 3m Al[TFSI]$_3$. The initial potential was -1.4 V, (B) Charge-discharge vs capacity profiles measured during Al plating/stripping at Zn/carbon cloth cell using 3m Al[TFSI]$_3$ and 2m $Al_2(SO_4)_3$ electrolytes at applied current density of 0.2 mA cm^{-2} and (C) XRD of Zn substrate after charge-discharge using asymmetrical Zn/carbon cell in 3m Al[TFSI]$_3$*

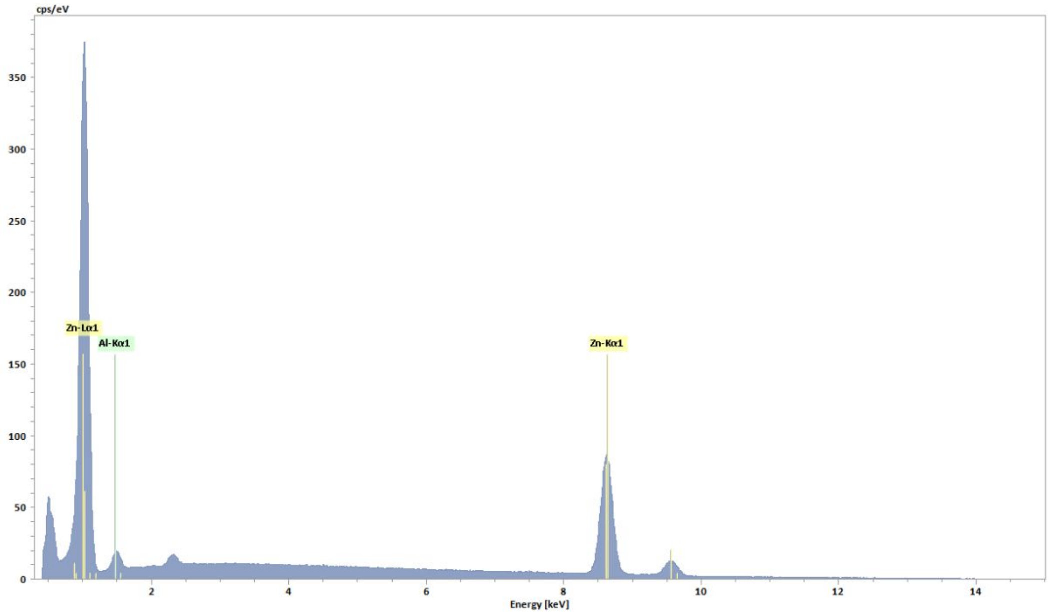

Figure S3 *Energy-dispersive X-ray spectroscopy of the Zn substrate obtained after the charge-discharge cycle in 3m Al[TFSI]$_3$*

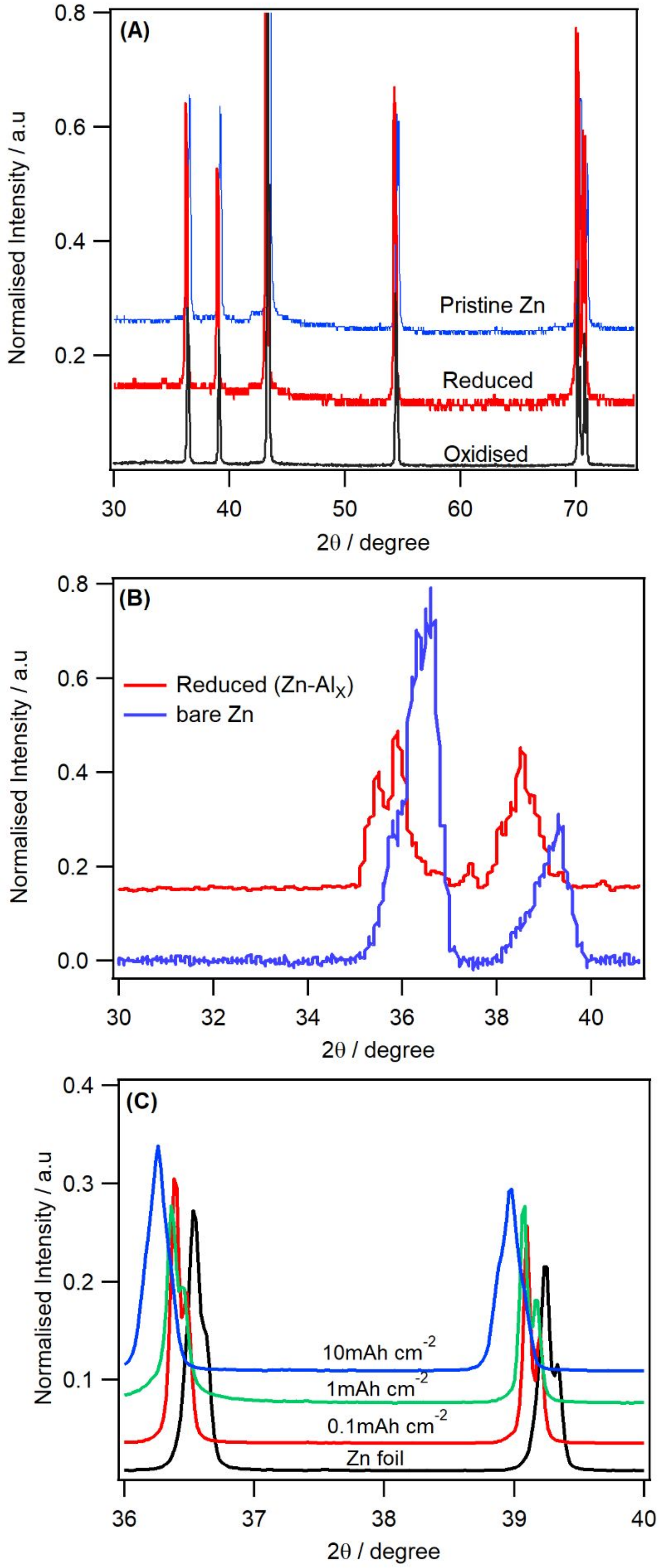

Figure S4 *(A), (B) XRD of Zn substrate after charge-discharge using symmetrical Zn/Zn cell in 3m Al[TFSI]$_3$ and (C) XRD of Zn substrate after charging at shown capacity using Zn/MnO$_2$ cell in 3m Al[TFSI]$_3$*

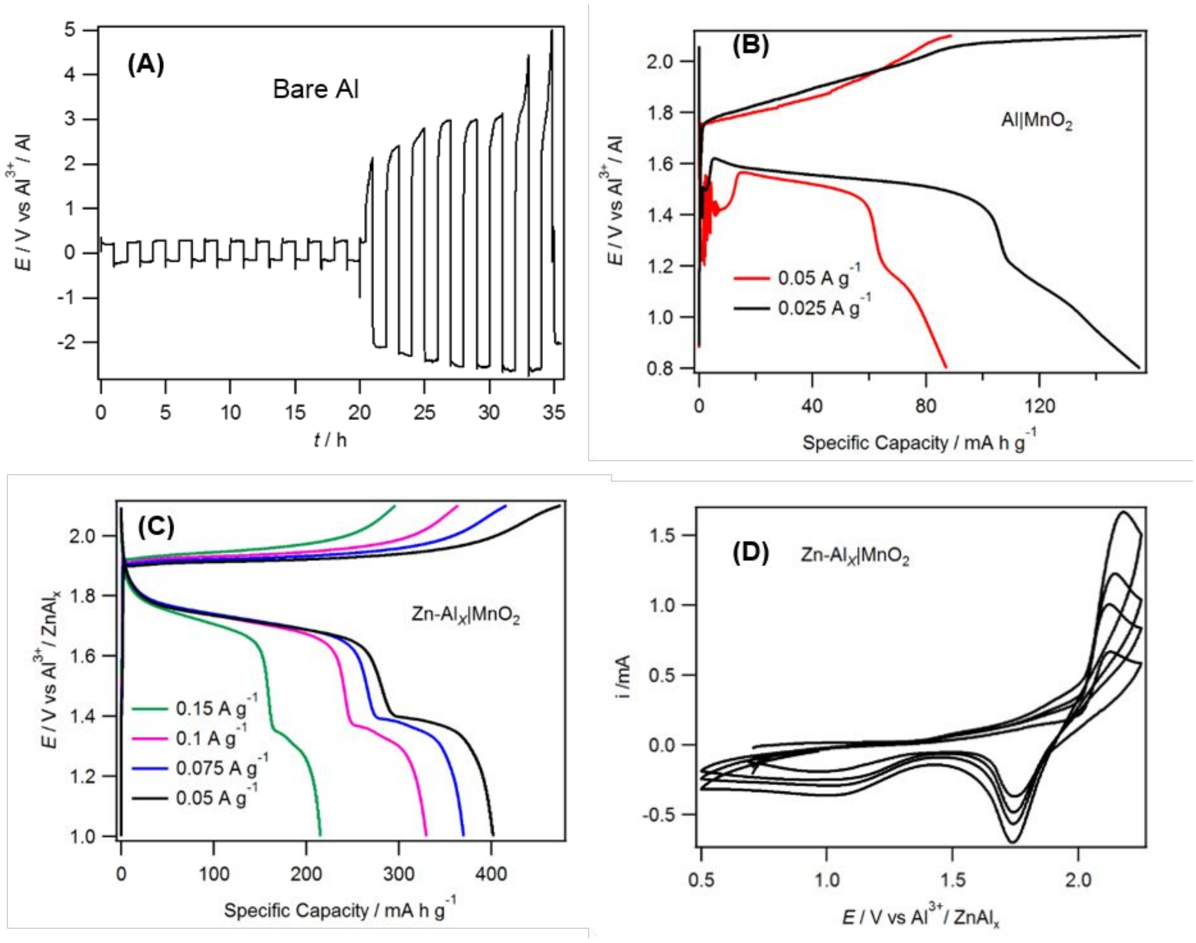

Figure S5 *(A)charge-discharge curve of symmetrical Al/Al cell in 3m Al[TFSI]*$_3$ *at 0.2 mA cm*$^{-2}$*, (B) Galvanostatic Charge-discharge curves vs capacity obtained using bare Al negative electrode and* MnO_2 *positive electrode in 3m Al[TFSI]*$_3$*, (C) Specific capacity of the cell at indicated current density during charge–discharge using Zn-Al*$_X$*/3m Al[TFSI]*$_3$*/*MnO_2 *cell and (D) Cyclic voltammograms recorded at 10, 5, 3 and 1 mV s*$^{-1}$ *(top to bottom) in 3m Al[TFSI]*$_3$ *electrolyte using coin cells constructed from* MnO_2 *positive electrodes and a Zn-Al*$_X$ *foil negative electrode*

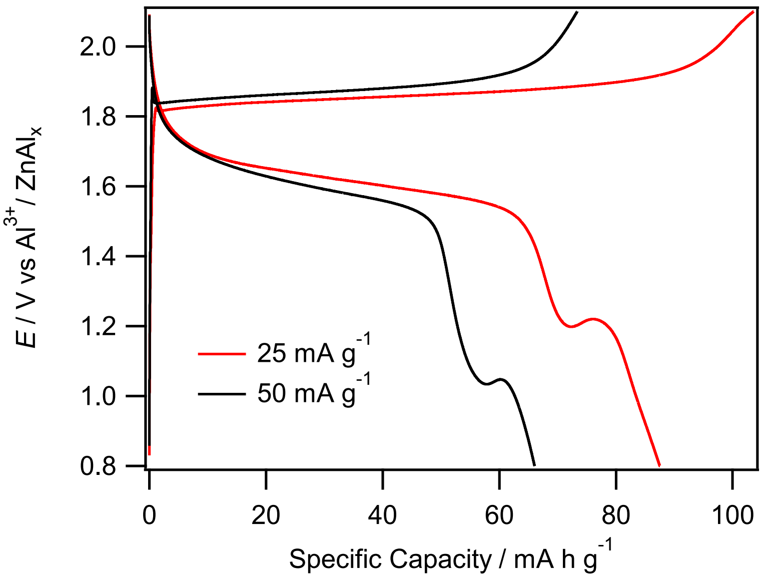

Figure S6 *Galvanostatic Charge-discharge curves vs capacity obtained using Zn-Al_X negative (pre-made) electrode and MnO_2 positive electrode in 3m Al(TFSI)$_3$.* The *Zn-Al_X was first made from the cell using Zn negative electrode and MnO_2 positive electrode by charging the cell to 2.15 V in 3m Al[TFSI]$_3$. The electrochemically treated Zn negative electrode was then rinsed in water and reused as the negative electrode.*

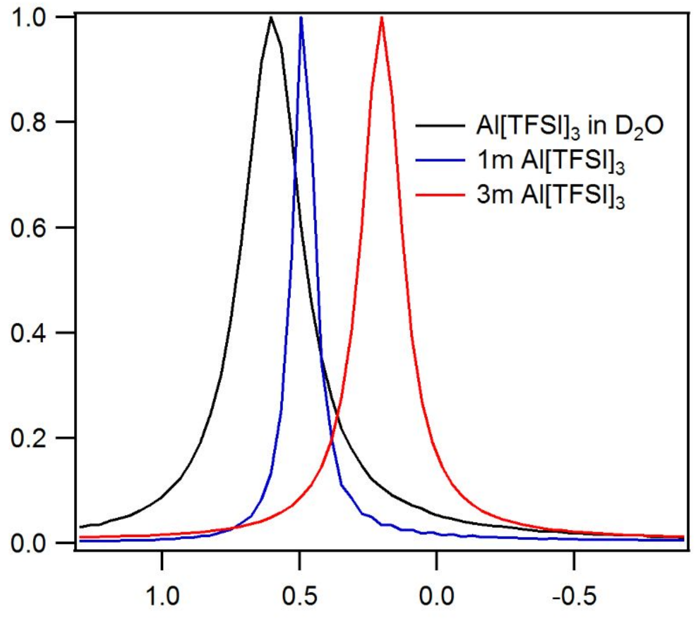

Figure S7. *Normalized NMR spectra of aqueous Al electrolytes showing ^{27}Al of 3m Al[TFS]$_3$ at shown concentration*

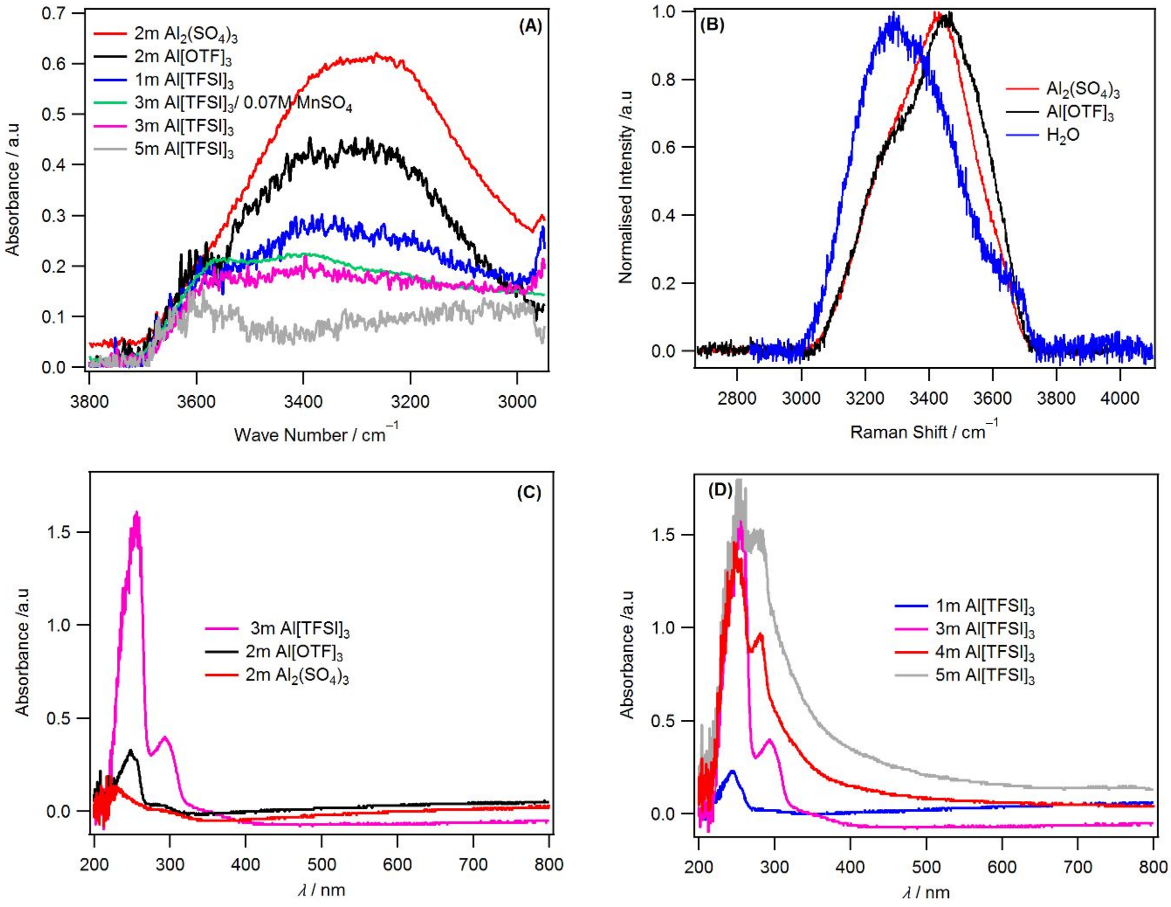

Figure S8 (A) *FTIR absorbance spectra of shown electrolytes, (B) Raman Spectra, (C) and (D) UV–vis spectra of the shown electrolytes*

Fourier-transform infrared spectroscopy (FTIR) and Raman spectroscopy were used identify the change in the solvation structure of the Al electrolytes (Figure S7). The characteristic water (O-H) stretching mode was seen at centered at ~3330 cm^{-1} for 1 m Al[TFSI]$_3$, 2m Al[OTF]$_3$ and 2m $Al_2(SO_4)_3$. However, with an increase in the concentration of Al[TFSI]$_3$ above 1m the intensity of the peak reduced and new peaks at higher wavenumbers appear indicating the presence of new environments for water molecules. The change in water environment explains the enhanced electrochemical stability of Al[TFSI]$_3$. This observation was consistent for LiTFSI solution when its concentration reached water-in-salt region.[5] FTIR also showed that the addition of $MnSO_4$ to Al[TFSI]$_3$ did not cause any measurable change in the structure of water molecules. Raman spectroscopy for pure water showed a broad Raman band between

3000 and 3700cm^{-1} for the OH stretching due to the diverse hydrogen-bonding environments.[6] With Al[OTF]$_3$ and 2m $Al_2(SO_4)_3$ the OH stretching position blue shifted by the rise of a broad peak at ~ 3450 cm^{-1}. Al[TFSI]$_3$-based salt is highly fluorescence so not able to get any useable result. UV–vis absorption spectra of 1m Al[TFSI]$_3$ electrolyte showed one broad absorbtion band at ~250 nm which are similar to 2 m Al[OTF]$_3$ electrolyte and $Al_2(SO_4)_3$(showed a small broad band at ~245 nm). A second absorbtion band appeared at ~295 nm for Al[TFSI]$_3$ as its concentration is increased, which might be due to the $\pi\rightarrow\pi^*$ excitation.[7] This band was seen for 3m Al[TFSI]$_3$ at 295 nm and then blue shifted to 283 nm for the 4m before it was merged with the other band for the 5m.

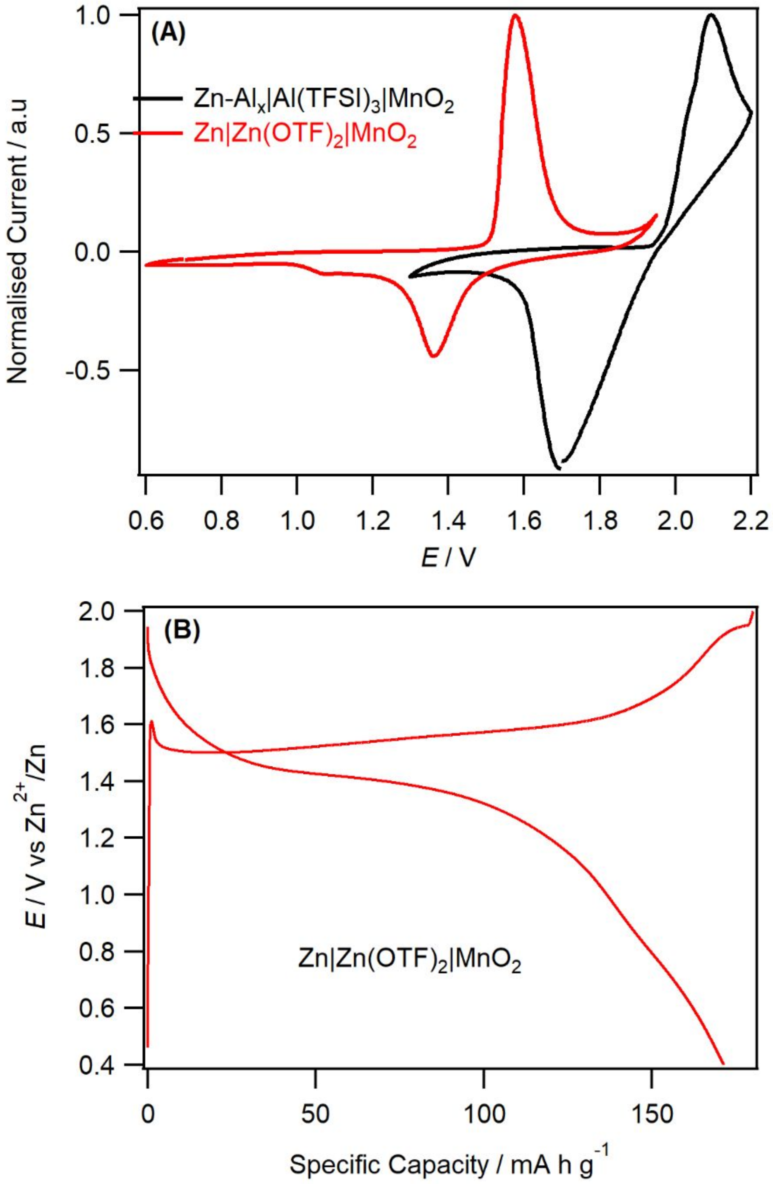

Figure S9. *(A) CV comparison recorded at 3m Al[TFS]$_3$ vs 3m Zn[OTF]$_2$ using shown positive and negative electrode and (B) Galvanostatic Charge-discharge curves vs capacity obtained at a current density of 100 mA g^{-1} using Zn negative electrode and MnO$_2$ positive electrode in 3m Zn[OTF]$_2$*

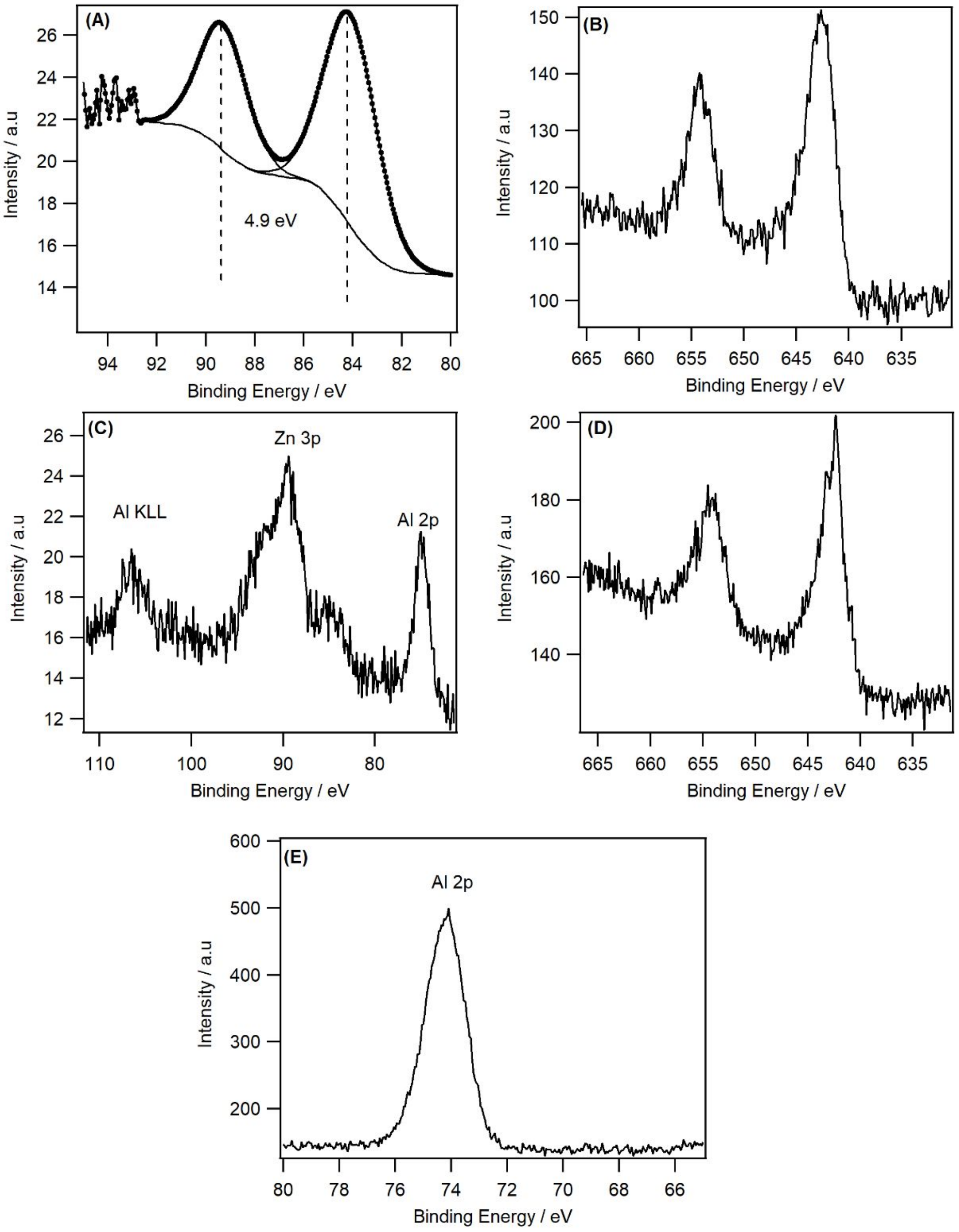

Figure S10 *(A) and (B) HAXPES of charged MnO_2 electrode in Mn 3s (A) and Mn 2p (B) region. (C) and (D) are XPS of discharged MnO_2 in Mn 3s and Mn 2p region. (E) XPS of Zn anode after fully charging the cell in Al 2p region.*

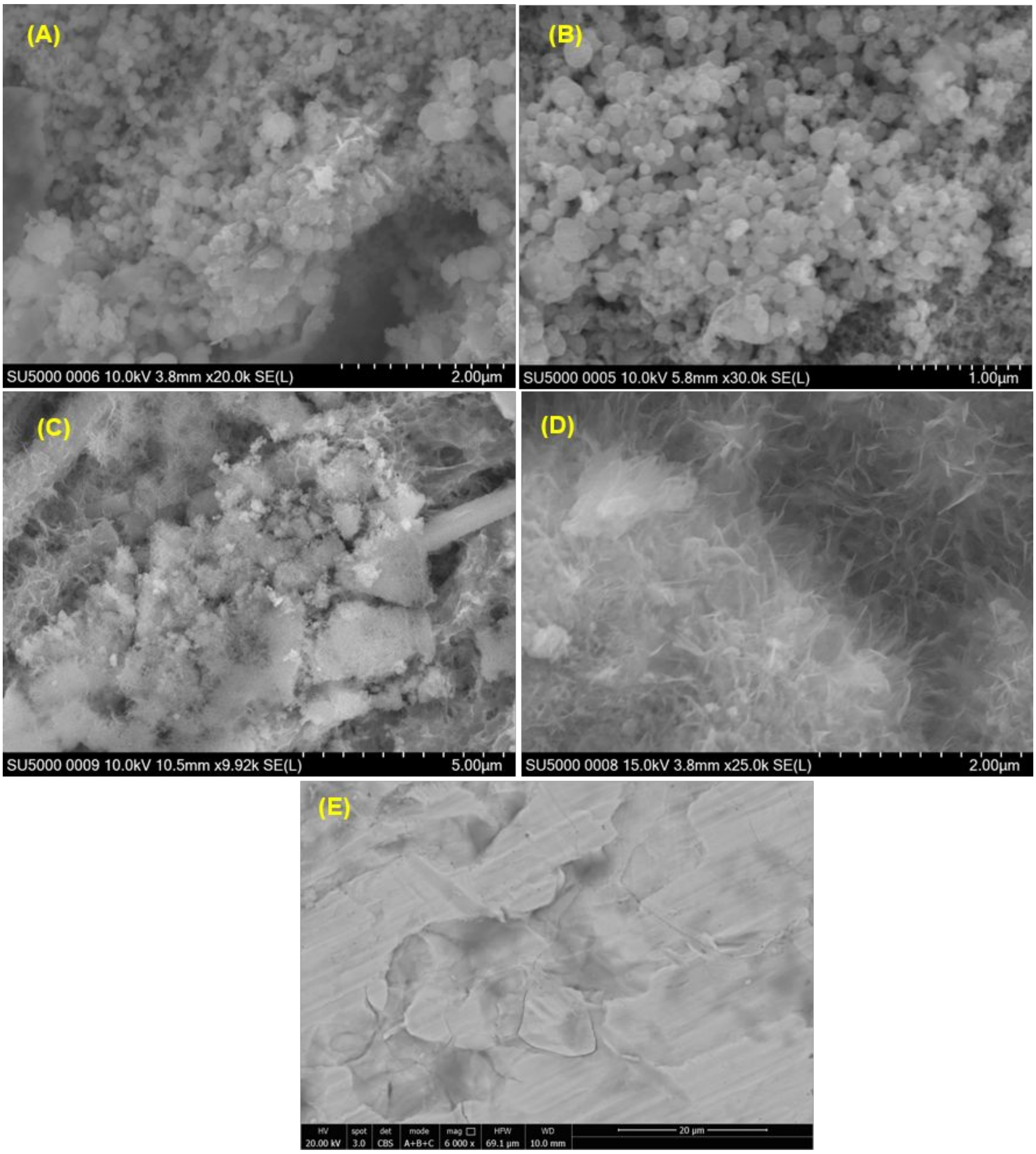

Figure S11 SEM images of the Zn-Al_X electrodes after charging in full cell using Zn foil negative electrodes and MnO_2 positive electrodes in (A-B) 3m Al[TFSI]$_3$ and (C-D) 5m Al[TFSI]$_3$ and (E) bare Zn

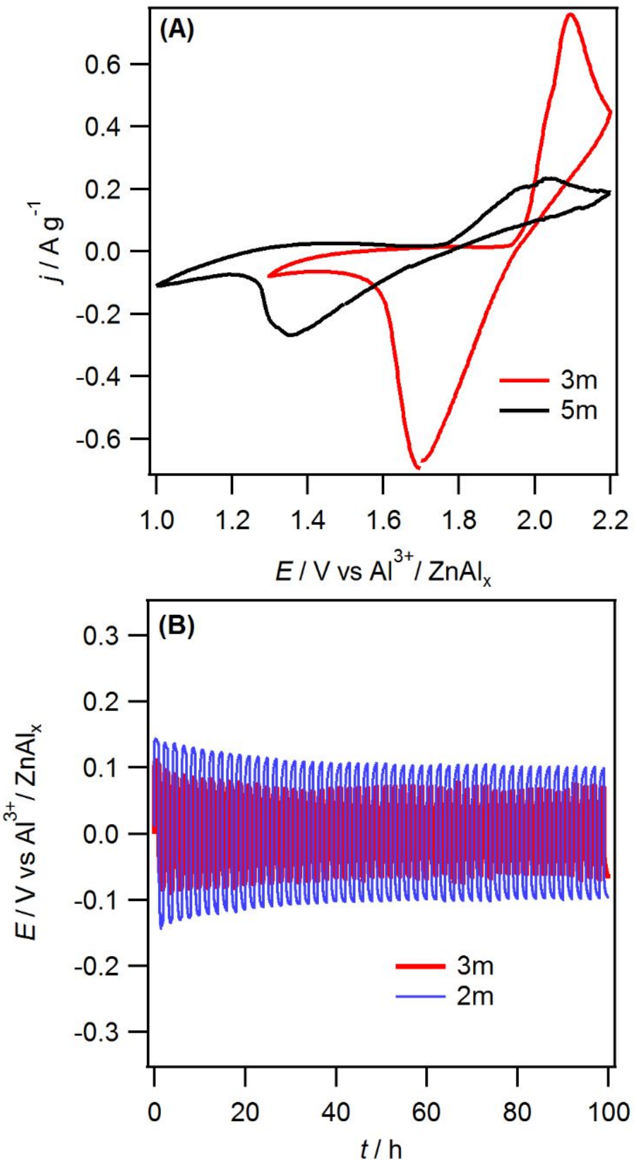

Figure S12 *(A) Cyclic voltammograms recorded at 0.1 mV* s^{-1} *in shown electrolyte using coin cells constructed from* MnO_2 *positive electrodes and a Zn-*Al_X *foil negative electrode and (B) Galvanostatic charge-discharge curve obtained using symmetrical Zn/Zn cell in 2.0 and 3.0 m Al[TFSI]*$_3$ *electrolytes at 0.2 mA* cm^{-2}

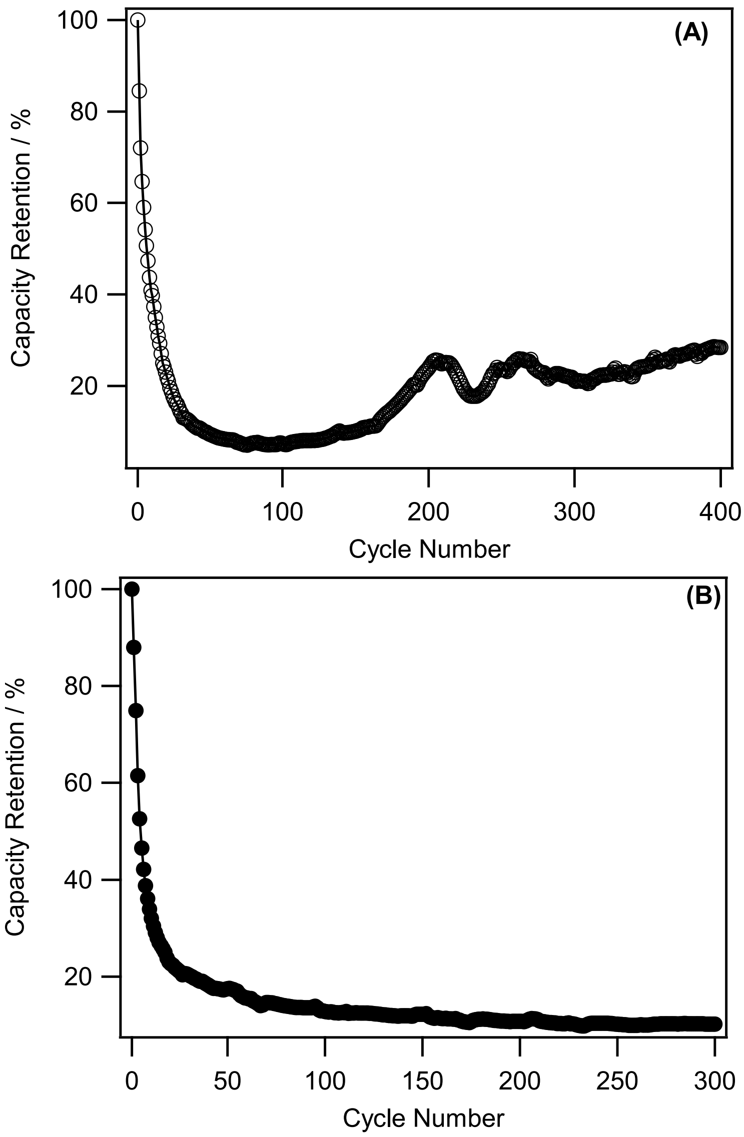

Figure S13*. Capacity retention and columbic efficiency of Zn-Al_X/MnO_2 cell obtained in (A) 2m Al[OTF]$_3$ and (B) 2m Al_2SO_4 each electrolyte contains 70mM $MnSO_4$ additives.*

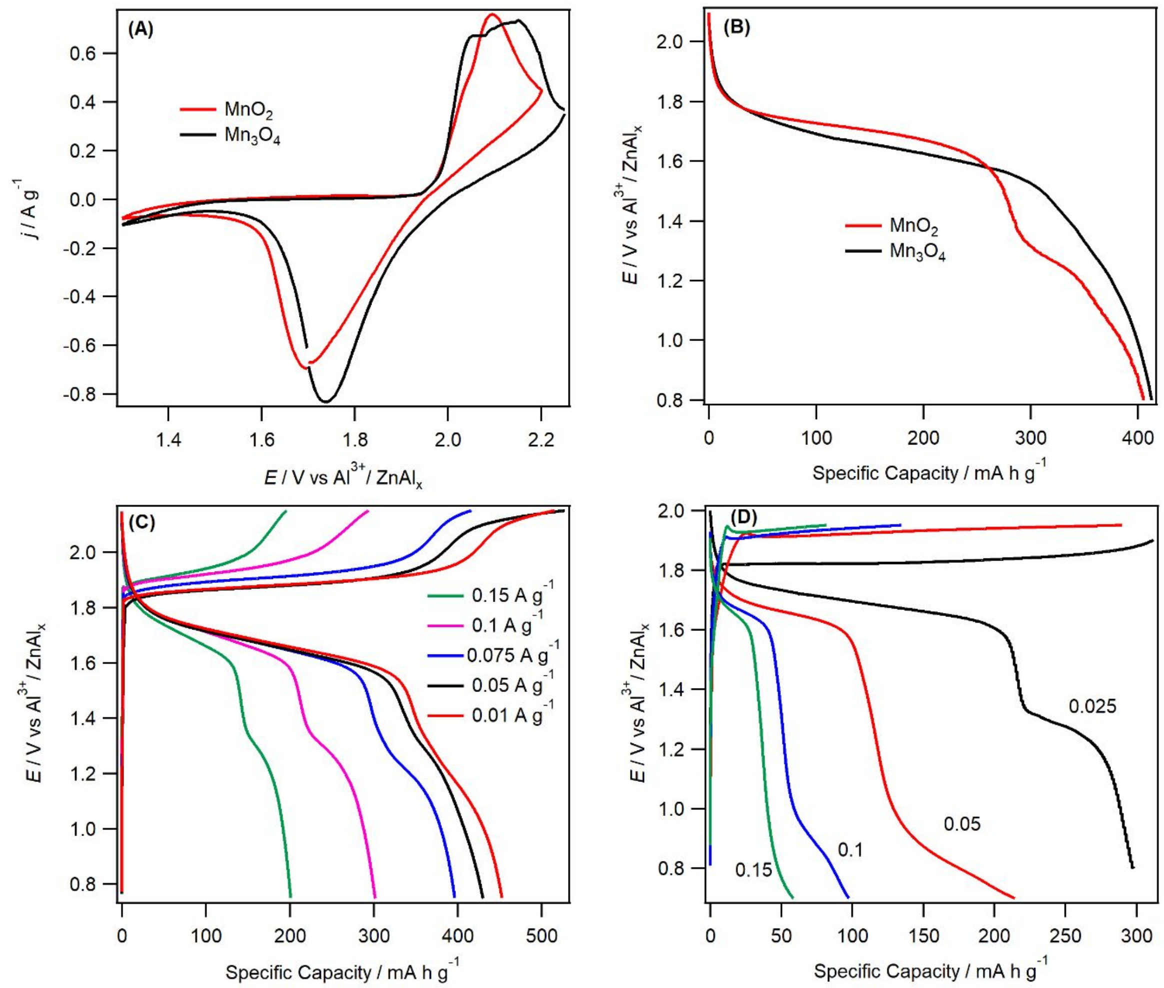

Figure S14 *(A) CVs recorded at 0.1 mV s^{-1} with the Mn_xO_y positive electrode (defined within the figure) using 3m Al[TFSI]$_3$, (B) Charge-discharge curves vs capacity obtained at a current density of 50 mA g^{-1} using Zn-Al_X negative electrode and the Mn_xO_y positive electrode in 3m Al[TFSI]$_3$, (C) Charge-discharge curves vs capacity obtained at indicated current density using 3m Al[TFSI]$_3$ and Mn_3O_4 positive electrode and Zn-Al_X negative electrode and (D) Charge-discharge curves vs capacity obtained at indicated current density (A/g) using 2m $A_2(SO_4)_3$ and α-MnO_2 positive electrode and Zn-Al_X negative electrode*
